# Linking ISO Dynamic Stiffness and Acoustic Modal Identification for FEM-Oriented Modelling of Elasticized Expanded Polystyrene

**DOI:** 10.3390/ma19163540

**Published:** 2026-08-20

**Authors:** Krzysztof Nering, Konrad Nering, Ewa Kozak-Jagieła

**Affiliations:** 1 Faculty of Civil Engineering, Cracow University of Technology, 31-155 Cracow, Poland; 2 Faculty of Mechanical Engineering, Cracow University of Technology, 31-155 Cracow, Poland; konrad.nering@pk.edu.pl; 3 Faculty of Environmental Engineering and Energy, Cracow University of Technology, 31-155 Cracow, Poland; ewa.kozak@pk.edu.pl

**Keywords:** elasticized expanded polystyrene, dynamic stiffness, floating floors, impact sound insulation, acoustic modal identification, finite element method, contact compliance, damping, Poisson’s ratio

## Abstract

**Highlights:**

- ISO stiffness and modal FEM give consistent EPS-T effective modulus.
- A series compliance term is needed to link E/h with ISO stiffness.
- Direct E/h conversion overestimates stiffness and lowers predicted Δ*Lw*.
- Damping depends on the test configuration and cannot be transferred directly.
- ISO s′ remains the preferred standardized input for EN ISO 12354-2 Annex C calculations within their stated field of application.
- Modal E can reproduce ISO-type stiffness only when contact/support compliance is included.
- EPS-T parameter sets should report interface and support assumptions.
- Further tests should separate material, contact and damping effects.

**Abstract:**

Elasticized expanded polystyrene (EPS-T) is widely used as a resilient layer in floating floors, where dynamic stiffness is the key input for impact-sound prediction, while FEM modelling additionally requires elastic parameters and damping descriptions consistent with the adopted test or modelling configuration. This study investigates whether ISO-type dynamic stiffness testing and acoustic-response modal identification can provide consistent vibroacoustic parameters for EPS-T. Rectangular specimens of different thicknesses were tested for dynamic stiffness and damping using an ISO 9052-1-type setup. Additional cylindrical compression tests were used to examine apparent Young’s modulus and Poisson’s ratio, while impulse-excited acoustic responses of clamped specimens were combined with inverse FEM identification. The ISO-type dynamic stiffness decreased from approximately 53.3 MN/m^3^ for nominal 17 mm specimens to 27.0 MN/m^3^ for nominal 53 mm specimens. This trend was described by a compliance model with an effective Young’s modulus of 2.04 MPa and an equivalent contact/support stiffness of 101.2 MN/m^3^. Acoustic-response inverse FEM gave consistent Young’s modulus values, ranging from 1.77 MPa to 2.16 MPa, with a mean close to 2.05 MPa. Direct use of *s*′ = *E*/*h* overestimated stiffness and underestimated predicted Δ*L_w_* by approximately 2–5 dB. The two routes provided consistent estimates of the effective modulus, but this consistency applies only to modulus identification and not to direct stiffness conversion or damping transfer.

## 1. Introduction

Progressive urbanization and the increasing density of multi-family residential buildings expose occupants to combined noise and vibration more frequently [1,2,3]. These stimuli may originate from road, tram or railway transport, but also from the operation of the building itself, including the activity of other occupants and technical equipment. They affect acoustic and vibration comfort and, under long-term exposure, may reduce the quality of life of building users [4,5,6,7]. Low-frequency phenomena are particularly important, because the dynamic response of building partitions may result both in perceptible vibration and in the radiation of structure-borne noise [6,8,9]. Therefore, effective mitigation requires the proper selection of vibroacoustic solutions and reliable knowledge of the material parameters controlling stiffness, damping and vibration transmission [10,11,12].

One of the most commonly used solutions for reducing impact sound transmission in residential buildings is the floating floor. Such a system usually consists of a loading layer, most often a screed, separated from the structural floor by a resilient layer. This limits the direct transmission of vibration from the usable floor layer to the building structure [13]. The solution is particularly attractive in practice because it provides a significant improvement in impact sound insulation with a relatively simple construction technology. In typical applications, the resilient layer is made of elasticized expanded polystyrene (EPS-T), which combines low dynamic stiffness, material availability and ease of installation [12,14,15]. From the point of view of dynamic behaviour, however, a floating floor is a mass–spring–damper system. This means that its effectiveness does not depend only on the presence of the resilient layer, but also on the proper selection of its stiffness and damping in relation to the mass of the loading layer and the expected operating frequency range of the system [16,17,18].

In the standard assessment of floating floors, the basic parameter of the resilient layer is the dynamic stiffness determined in accordance with ISO 9052-1 [19]. In engineering models, such as EN ISO 12354-2 [20], the reduction in impact sound level can be estimated mainly from the dynamic stiffness of the resilient layer and the surface mass of the loading layer. This approach is practical and well established for typical floating-floor systems, because the stiffness of the resilient layer determines the resonance frequency of the mass–spring system [16,18,21]. At the same time, damping affects the response amplitude near resonance, which is particularly important in the low-frequency range [17,22,23]. For more complex geometries, local discontinuities or detailed analyzes of the dynamic response, FEM modelling becomes necessary [24,25,26]. Such a model, however, requires parameters that go beyond dynamic stiffness alone, in particular, Young’s modulus, Poisson’s ratio and a damping description consistent with the adopted numerical configuration [27,28,29,30]. Damping determined from the resonance response of the ISO 9052-1 [19] system should therefore not be treated as a direct material parameter, because it also includes the effects of contact, support conditions and the test configuration itself.

Although ISO 9052-1 dynamic stiffness is sufficient for standard impact-sound calculations, FEM models require Young’s modulus, Poisson’s ratio and a damping description consistent with the numerical configuration. The central question is therefore whether the standard dynamic stiffness of EPS-T can be linked to these FEM-oriented parameters without losing the physical meaning of the ISO result [19,28,31,32,33,34,35,36].

Converting the dynamic stiffness obtained from the ISO 9052-1 test into FEM material parameters is not straightforward [30,37,38,39]. A direct interpretation in the form of *s*′ = *E*/*h* neglects the effect of Poisson’s ratio and assumes that the measured compliance results only from material deformation [30,40]. In practice, the test result may also include contact compliance, support conditions and local deformation effects in the specimen [38,41]. A similar problem applies to damping, because the value determined from the resonance response describes the whole measurement system rather than the material property alone [27,42]. Therefore, the transition from *s*′*_ISO_* to *E*, *ν* and damping requires additional interpretation, not a simple conversion of the standard test result [39,40].

Alternative test methods can complement ISO 9052-1 when parameters for FEM modelling are required [43,44,45,46]. This applies in particular to mechanical tests on specimens with different geometries, dynamic response analysis and inverse identification using numerical models [34,47,48]. These methods can provide Young’s modulus, Poisson’s ratio or modal damping, but they should not be treated as direct substitutes for ISO 9052-1 [19,45,49]. It is more appropriate to use them as a bridge between the standard acoustic characterization and the material parameters required in FEM [50,51,52].

Previous studies have addressed ISO dynamic stiffness [19], contact-sensitive mechanical identification [38,41] and FEM-oriented parameter estimation [34,47,48] separately. However, their consistency for the same EPS-T product family, including the compliance required to link the effective modulus with ISO-type dynamic stiffness, has not been experimentally demonstrated.

**Figure 1:**
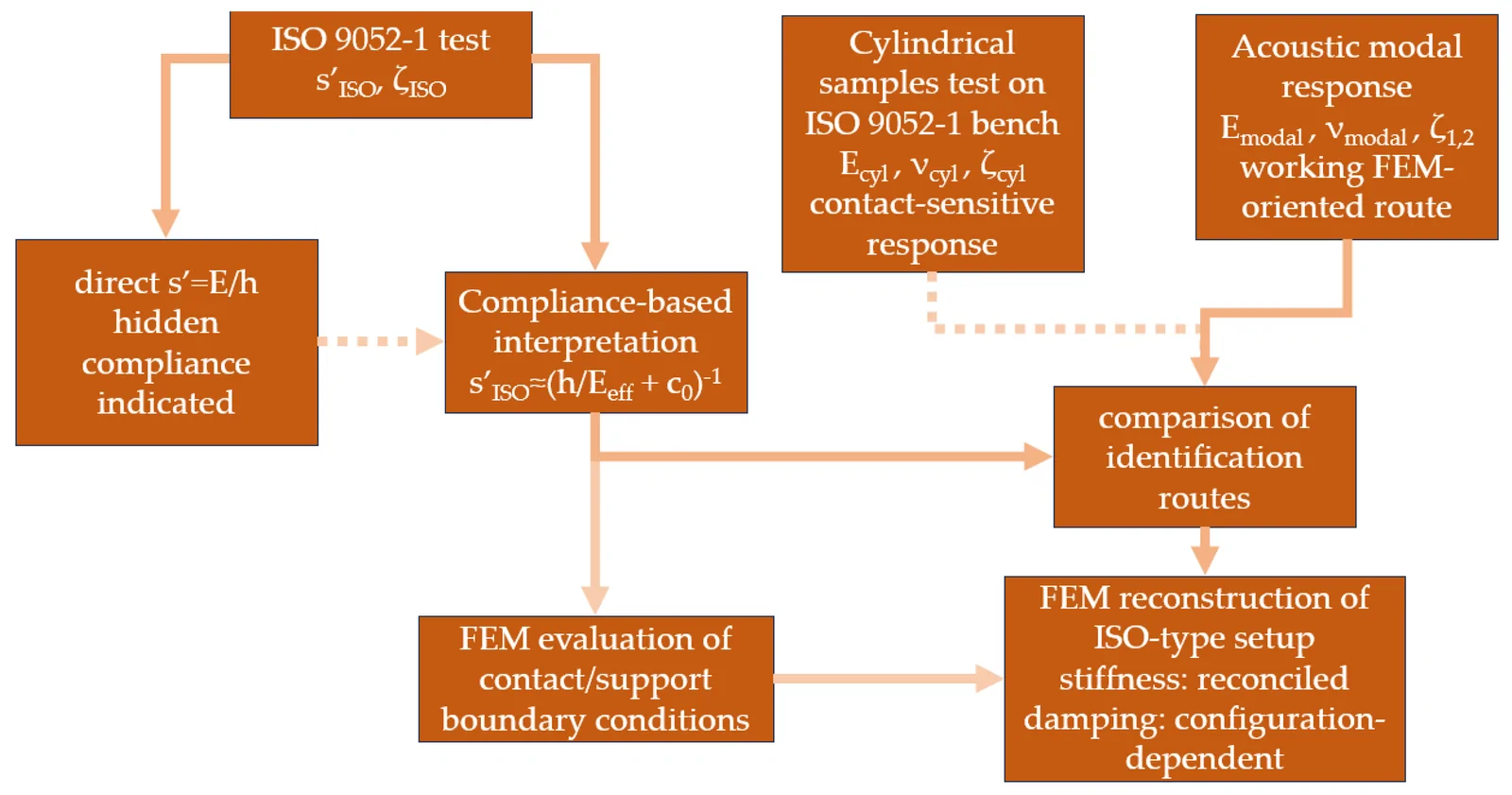
Conceptual map of the identification strategy used in this study. The scheme shows the transition from ISO 9052-1 dynamic stiffness to FEM-oriented elastic parameters, including the role of contact/support compliance and the configuration-dependent character of damping.

The conceptual logic of the study is summarized in Figure 1.

The aim of this study was to determine whether FEM-oriented parameters of elasticized expanded polystyrene (EPS-T) can be identified consistently with its standard ISO 9052-1 [19] acoustic characterization. ISO-type dynamic stiffness was used as the acoustic benchmark, while cylindrical compression tests [28,53] and impulse-excited acoustic modal response were used as complementary identification routes. The study specifically examined whether the direct relation *s*′ = *E*/*h* is sufficient for linking the effective Young’s modulus with ISO-type dynamic stiffness, or whether an additional contact/support compliance term is required. The novelty of the work lies in showing that ISO-compliance interpretation and acoustic-response inverse FEM can provide consistent estimates of the effective modulus only when this additional compliance is treated explicitly. At the same time, cylindrical compression is treated as a supporting diagnostic route, and damping is interpreted as configuration-dependent rather than as a directly transferable material constant.

## 2. Materials and Methods

### 2.1. Test Programme

The test programme included ISO 9052-1 [19] dynamic stiffness measurements, half-power damping analysis, frequency-response tests on cylindrical specimens with different aspect ratios, and impulse-excited acoustic-response measurements. These tests were used to estimate Young’s modulus, Poisson’s ratio and configuration-dependent damping.

**Figure 2:**
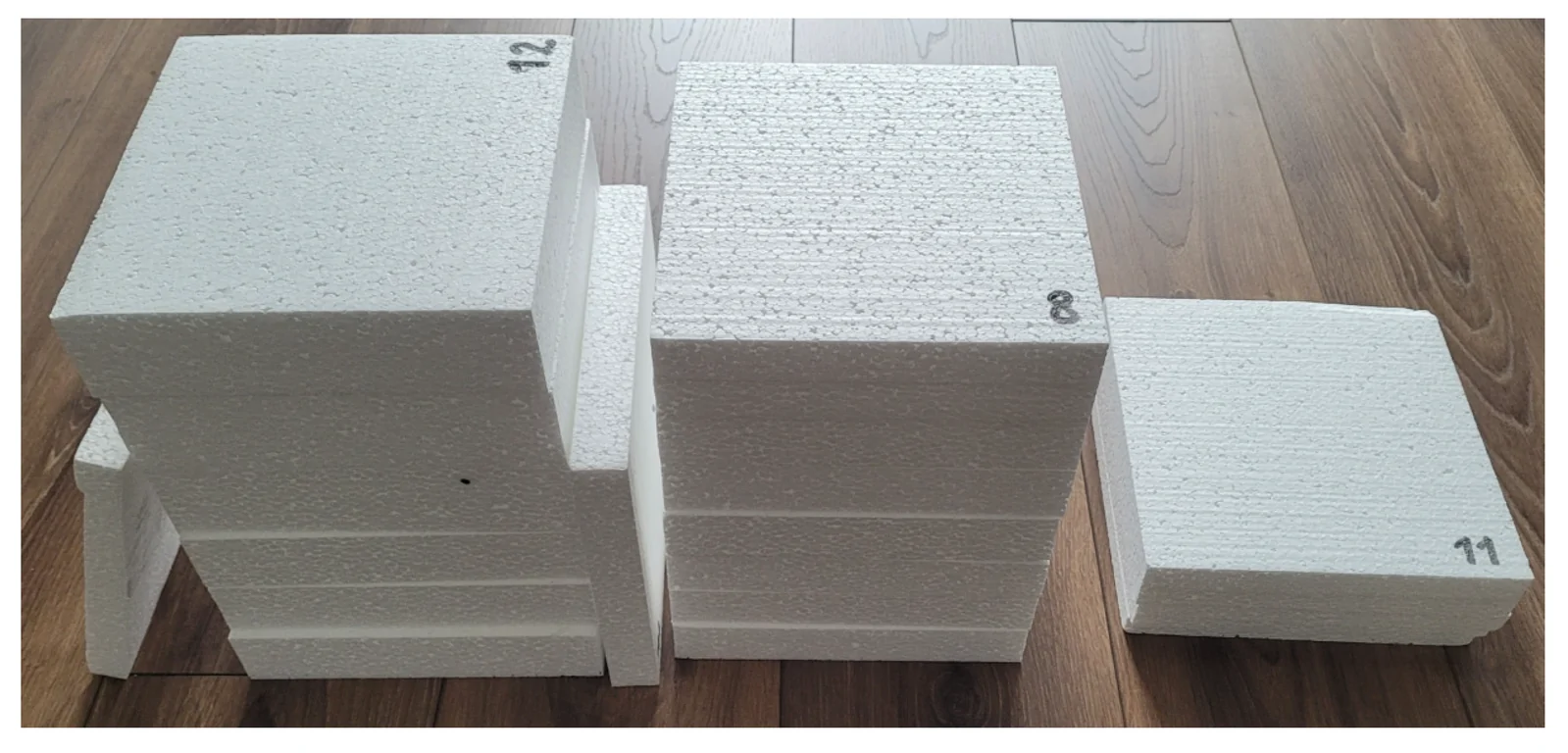
Rectangular specimens of resilient expanded polystyrene (EPS-T) with different thicknesses used in the dynamic stiffness and acoustic-response tests.

Rectangular specimens with in-plane dimensions of 200 × 200 mm were used for the dynamic stiffness tests and acoustic-response measurements. The specimens were prepared in accordance with ISO 9052-1 [19] and had different thicknesses of 17, 22, 27, 33, 38, 43, and 53 mm. The tested specimens were prepared from Termo Organika Sp. z o.o. (Kraków, Poland) EPS-T. All specimen thicknesses represented the same EPS-T product family and were obtained from the same production batch. Using a single production batch reduced within-study material variability but did not account for possible differences between batches. Therefore, the identified mechanical and vibroacoustic parameters should be regarded as representative of the tested batch and should be verified for other production batches. The measurements were performed at a temperature of 22.1 °C (CI95% 21.8; 22.6) and a relative humidity of 45.1% (CI95% 42.2; 52.5). The same set of specimens was used both for dynamic stiffness measurements and for acoustic-response tests under impulse excitation. Test samples are presented in Figure 2.

**Figure 3:**
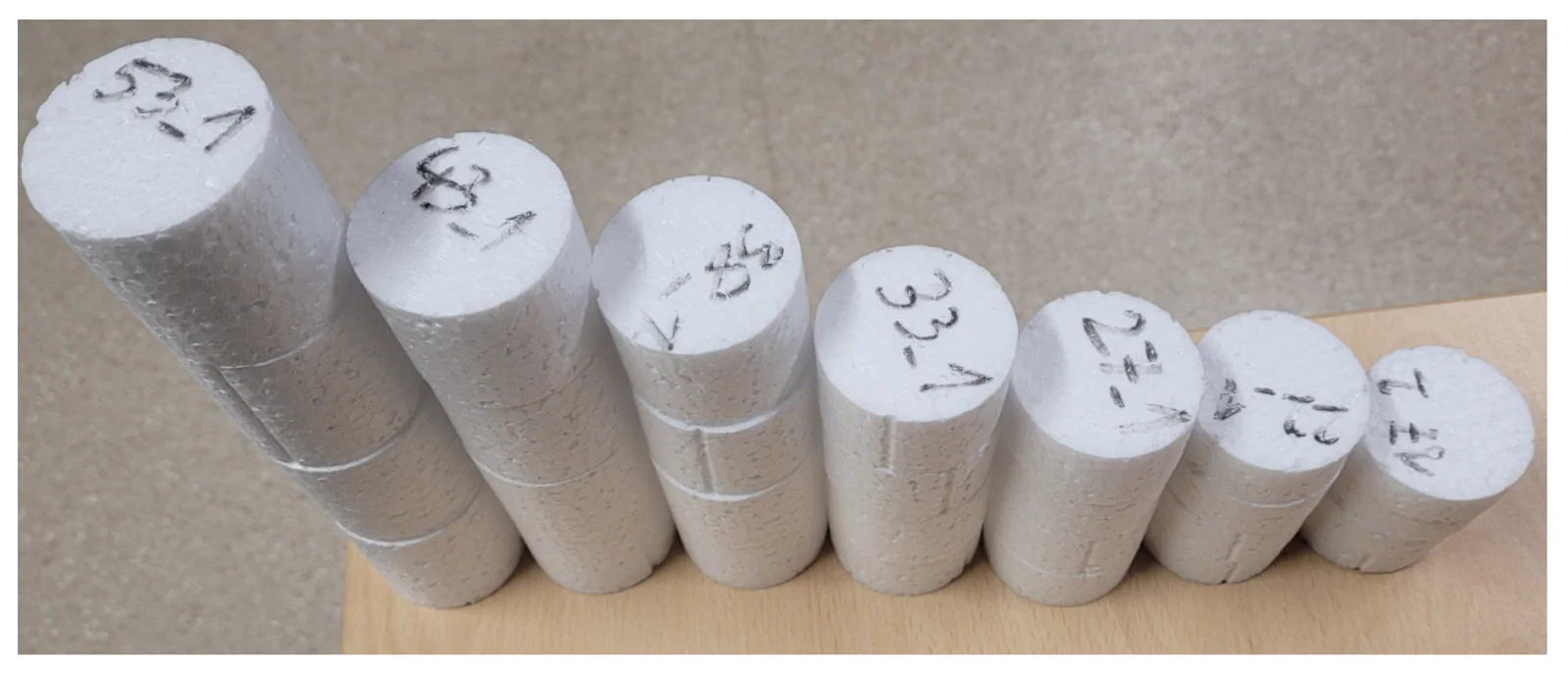
Cylindrical EPS-T specimens with different heights used for apparent Young’s modulus and Poisson’s ratio estimation.

Additional tests were performed on cylindrical specimens with a diameter of 50 mm and the same nominal heights as the rectangular specimens. These tests were used as a supporting diagnostic route to examine the sensitivity of compression-based identification to specimen shape, lateral constraint and contact conditions. They were not treated as the primary source of FEM-ready elastic parameters. Test samples are shown in Figure 3.

### 2.2. Dynamic Stiffness and Damping Test

Dynamic stiffness was measured using a test bench configured based on ISO 9052-1 [19]. The main components of the test bench are listed in Table 1.

The loading plate was selected so that the total moving mass, including the mounted measurement equipment and an approximately 5 mm plaster layer, was about 8.0 kg, corresponding to a static loading pressure of approximately 2 kPa. A separating foil was placed between the specimen and the loading plate. A plaster-of-Paris layer with a thickness of approx. 5 mm was used to compensate for local surface irregularities and improve contact uniformity. The test system was excited by a sinusoidal force generated by an electrodynamic shaker. The force amplitude was kept constant at 0.4 N with a tolerance of ±0.005 N. During the measurement, the excitation frequency was swept from 20 Hz to 350 Hz with a resolution of 0.1 Hz and a sweep rate of 0.1 Hz/s. The dynamic response of the system was recorded using an IEPE accelerometer mounted on the loading plate.

**Figure 4:**
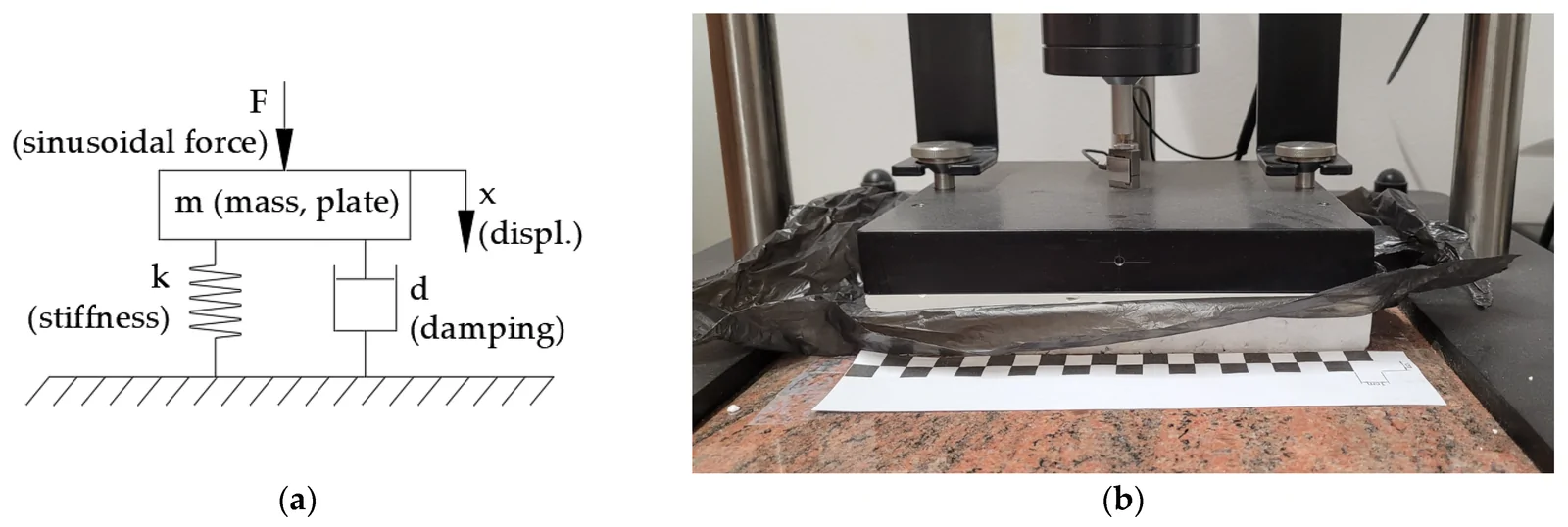
(**a**) Single degree of freedom model used to approximate the dynamic behaviour of the test bench. (**b**) Dynamic stiffness test bench—one square is equal to 1 cm [41].

The physical model of the test bench is presented in Figure 4.

The test bench enabled the frequency-response spectrum of the tested system to be obtained. The response spectrum was then used to determine the resonance frequency and the critical damping factor. Following ISO 9052-1 [19], the dynamic stiffness per unit area was determined from the measured resonance frequency as:

(1) $$s^{\prime }=4\pi^{2}m_{test}^{\prime }f_{r}^{2}$$

where *s*′—dynamic stiffness [MN/m^3^], *m*′*_test_*—mass per unit area in test [kg/m^2^], and *f_r_*—resonant frequency [Hz].

Because Equation (1) depends directly only on the total moving mass and resonance frequency, the amplitude uncertainties of the accelerometer, force sensor and excitation system were not propagated as separate terms. These components were used to identify the resonance peak and maintain stable linear excitation. Assuming independent inputs, the relative uncertainty was estimated from $u_{r\left(s^{\prime }\right)}=\sqrt{u_{r}^{2\left(m_{test}^{\prime }\right)}+4u_{r}^{2\left(f_{r}\right)}}$. The assumed plaster-thickness variation of 4–6 mm corresponds to a moving-mass variation of approximately ±0.047 kg. Together with the loading-plate weighing uncertainty and the 0.1 Hz frequency resolution, this resulted in an expanded relative uncertainty below 1% (*k* = 2), conservatively reported as approx. 1%.

The damping ratio was estimated using the half-power bandwidth method implemented by the authors in MATLAB R2026a Update 2 (26.1.0.3251617). The raw measurement data consisted of acceleration values recorded by the accelerometer.

Before damping estimation, the measured acceleration spectrum was converted into a pseudo-displacement spectrum using the frequency-domain relationship previously applied in dynamic-response testing [28,41,46]:

(2) $$\left|X(f)\right|=\frac{\left|A\left(f\right)\right|}{4\pi^{2}f^{2}}$$

where |*X*(*f*)|—pseudo-displacement spectrum [m], |*A*(*f*)|—acceleration spectrum [m/s^2^], and *f*—frequency [Hz].

**Figure 5:**
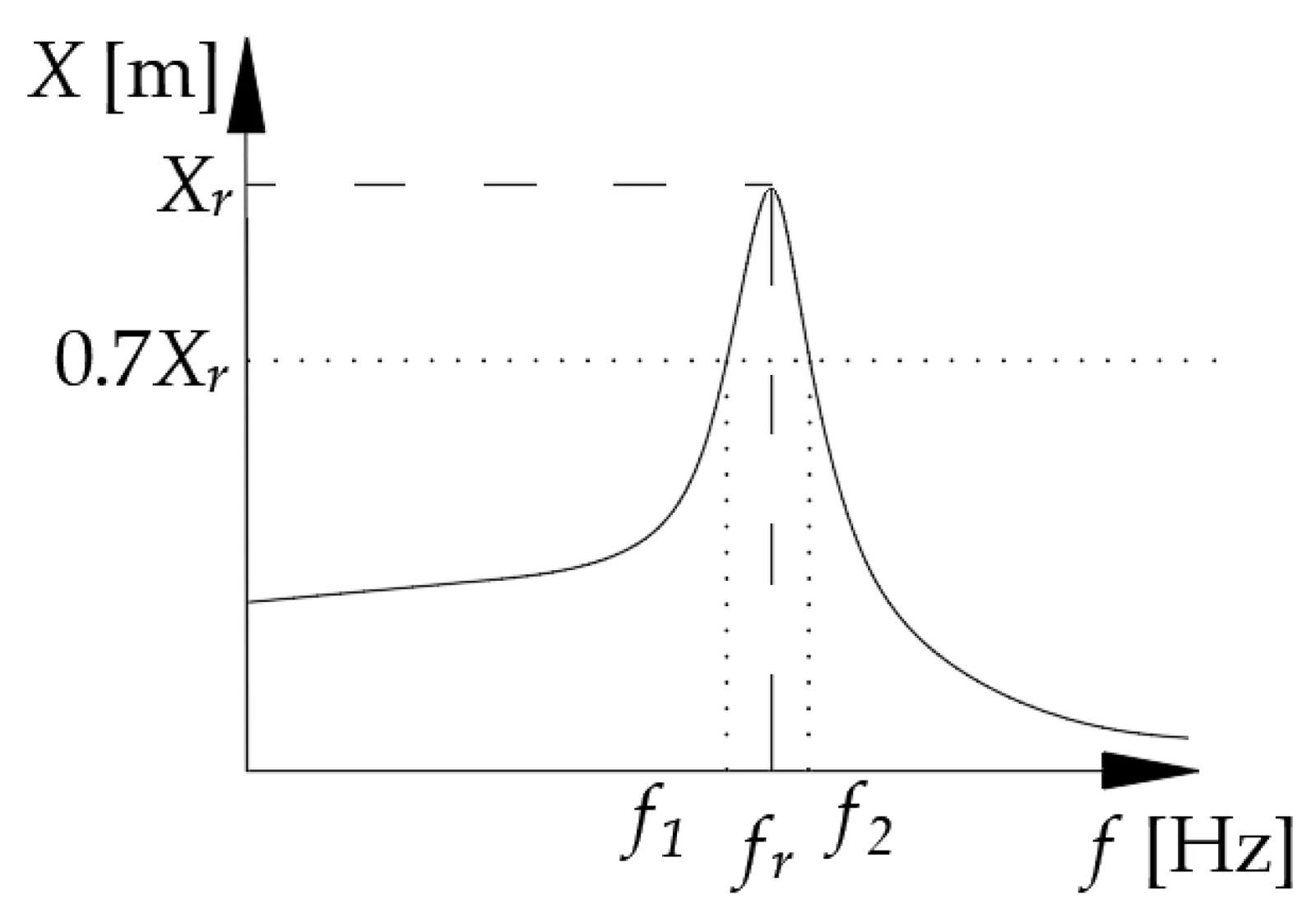
Schematic representation of the half-power bandwidth method based on the displacement response spectrum. *X_r_* denotes the displacement amplitude at the resonance frequency *f_r_*, whereas f_1_ and f_2_ correspond to 0.7 *X_r_*. The procedure is based on [28,41,46].

The half-power bandwidth procedure used for damping identification is illustrated schematically in Figure 5 [28,41,46].

The half-power frequencies *f*_1_ and *f*_2_ were then used to determine the logarithmic decrement and the corresponding critical damping ratio according to the half-power bandwidth relationships [28,41,46]:

(3) $$\frac{f_{2}-f_{1}}{f_{r}}=\frac{\delta }{\pi \sqrt{1-{\left(\frac{\delta }{2\pi }\right)}^{2}}}$$

(4) $$\delta =\frac{2\pi \zeta }{\sqrt{1-\zeta^{2}}}$$

### 2.3. Frequency Response Tests on Cylindrical Specimens for Apparent Young’s Modulus and Poisson’s Ratio Estimation

**Figure 6:**
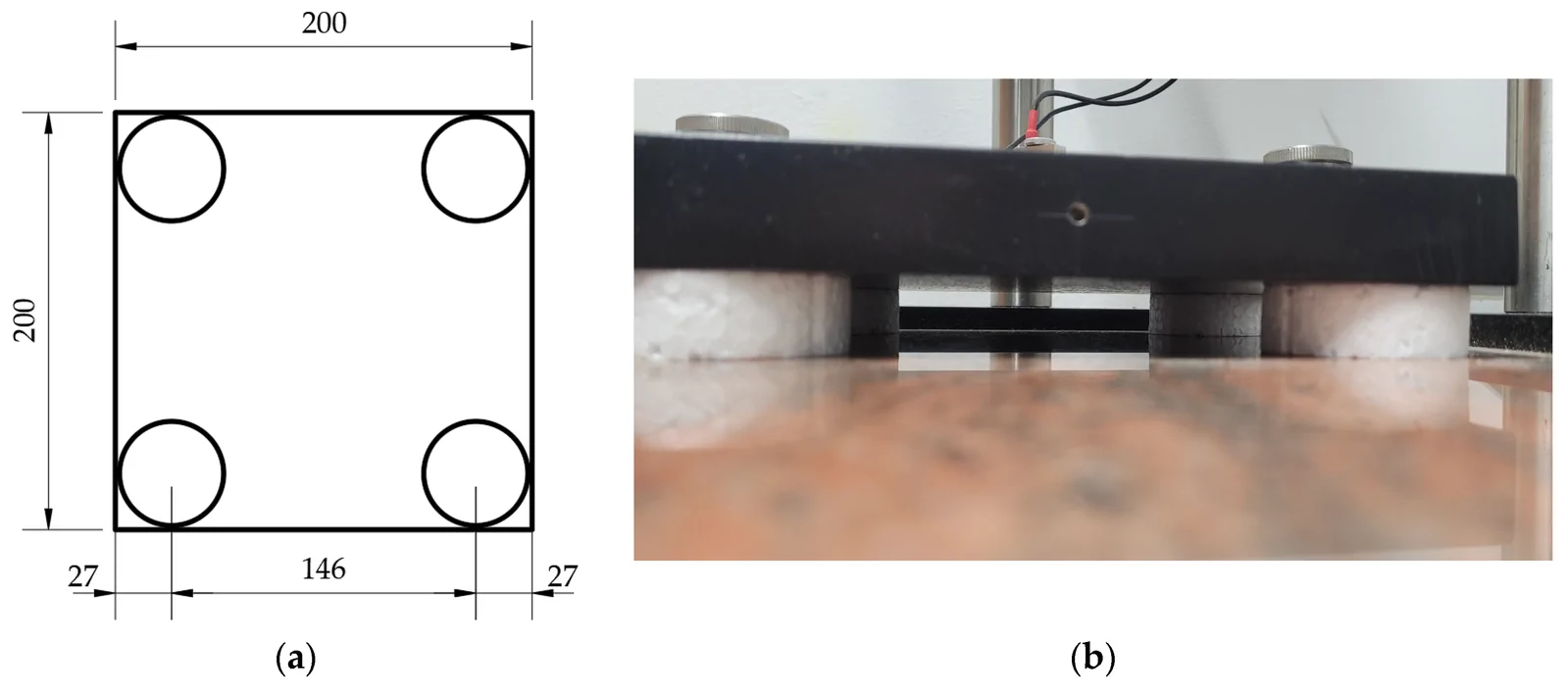
Symmetrical arrangement of four cylindrical EPS-T specimens (diameter of 50 mm) used in the dynamic compression test: (**a**) specimen layout under the 200 × 200 mm loading plate; (**b**) experimental setup with specimens placed between the rigid base and loading plate.

Four cylindrical specimens were tested simultaneously in the dynamic stiffness test bench. They were arranged symmetrically under the loading plate to ensure uniform load distribution and minimize system eccentricity. The excitation procedure was the same as for the dynamic stiffness and damping tests. For each specimen height, four repeated tests were performed with rotated specimen positions, and the final result was calculated as the mean value. Test setup is presented in Figure 6.

For the cylindrical specimens, the measured resonance frequency was converted into the total dynamic stiffness of the four-specimen system using the single-degree-of-freedom relationship applied in previous low-stress dynamic compression studies [28,41]:

(5) $$k_{tot}=m{\left(2\pi f_{r}\right)}^{2}$$

where *m*—the total moving mass of the loading system, *f_r_*—the resonance frequency of the tested system. Because the four specimens supported the same rigid loading plate and were arranged symmetrically, they were treated as parallel elastic elements. The dynamic stiffness of one cylindrical specimen was therefore obtained as [28,41]:

(6) $$k_{cyl}=\frac{k_{tot}}{n}$$

where *n* = 4 is the number of cylindrical specimens tested simultaneously. This interpretation assumes a piston-like motion of the loading plate and an approximately uniform load distribution between the cylindrical specimens.

The apparent dynamic Young’s modulus of an individual specimen was then calculated from its axial stiffness and geometry [28,41]:

(7) $$E_{a}=\frac{k_{cyl}h}{A_{cyl}}$$

where h is the specimen height and *A_cyl_* is the base area of the cylindrical sample.

The cylindrical-specimen aspect ratio was defined consistently with the previous low-stress compression methodology [53] as:

(8) $$S=\frac{r}{h}$$

The dependence of the apparent modulus on specimen geometry and Poisson’s ratio was subsequently described using the analytical relationship proposed by Williams and Gamonpilas [53]:

(9) $$E_{a}(E,\nu ,\;S)=E\frac{1+3\nu \left(\frac{1-\nu }{1+\nu }\right)S^{2}}{1+3\nu \left(1-2\nu \right)S^{2}}$$

where *E_a_* is the apparent Young’s modulus, *E* is the effective Young’s modulus of the material, *ν* is Poisson’s ratio, and S is the aspect ratio of the specimen. The parameters *E* and *ν* were identified simultaneously by nonlinear least-squares fitting of the model to the measured *E_a_*(*E*,*S*) data. In this approach, no specimen was assumed a priori to represent the unconstrained material modulus; instead, the material modulus and Poisson’s ratio were both obtained directly from the fitted curve.

The fitting procedure was performed in MATLAB R2026a Update 2 (26.1.0.3251617) using constrained nonlinear least squares, with (*E* > 0) and ν bounded within the physically admissible range selected for the analyzed material.

The fitted compression-based *E* and *ν* values were therefore interpreted as diagnostic parameters of the cylindrical test configuration rather than as final FEM material constants. The primary FEM-oriented elastic parameters were obtained from the acoustic-response inverse FEM procedure, whereas the cylindrical results were used to assess the sensitivity of compression-based identification to specimen geometry, contact effects and parameter identifiability.

### 2.4. Acoustic Response of Cantilever-like Specimens Under Impulse Excitation and FEM-Based Inverse Identification

**Figure 7:**
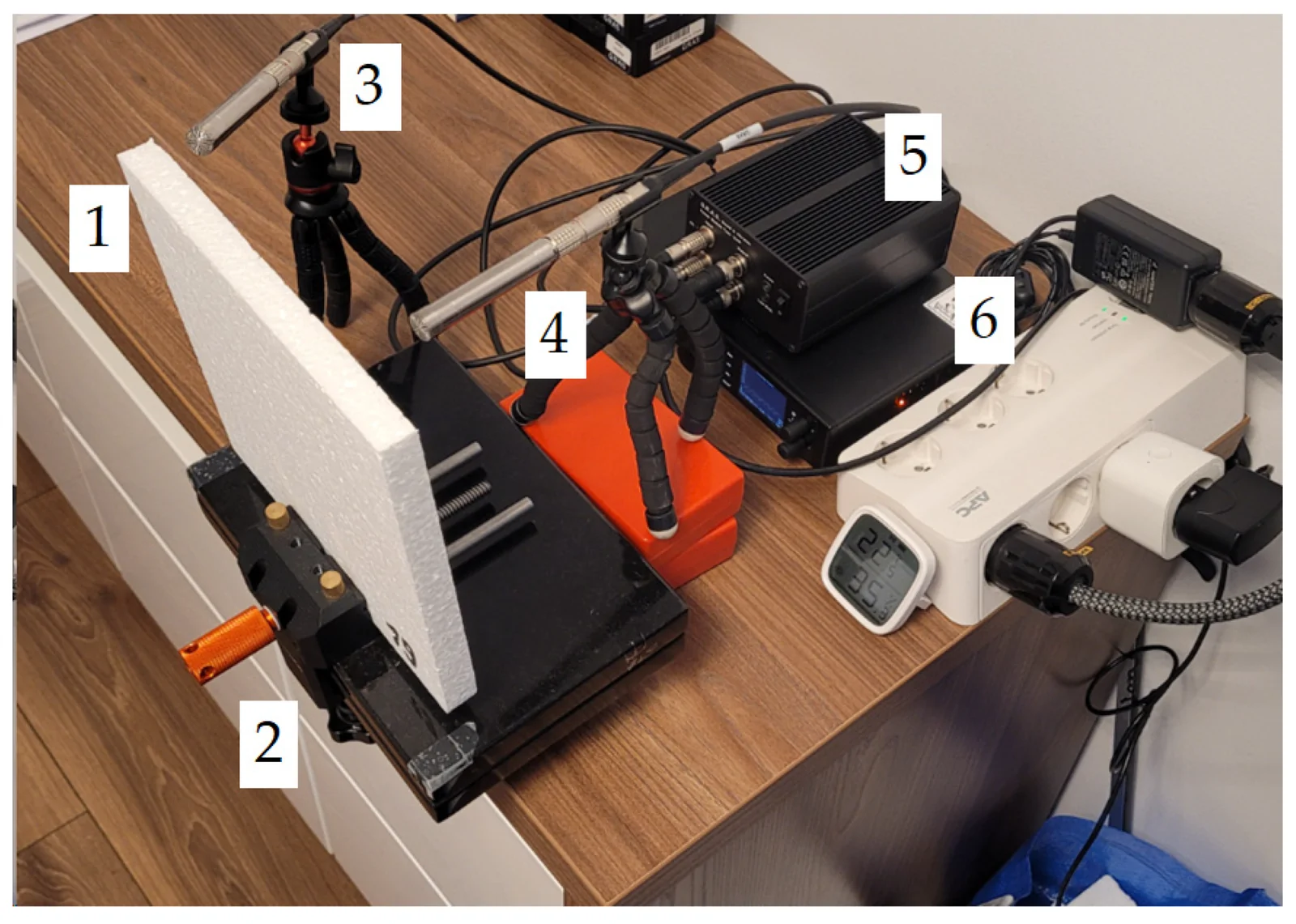
Experimental setup for impulse excitation testing of the clamped EPS-T specimen: (1) EPS-T specimen; (2) bench vise and clamping region; (3) microphone 1; (4) microphone 2; (5) GRAS 12AR two-channel power module; and (6) RME ADI-2/4 Pro SE audio interface and A/D converter.

Rectangular EPS-T specimens were also tested under impulse excitation to identify resonance frequencies and damping for bending- and torsion-dominated modes. Each specimen was clamped in a bench vise in a cantilever-like configuration and excited manually with a small impact hammer. The radiated acoustic response was recorded by two nearby measurement microphones to improve mode detection. The measurement equipment is listed in Table 2 and the experimental setup is shown in Figure 7.

**Figure 8:**
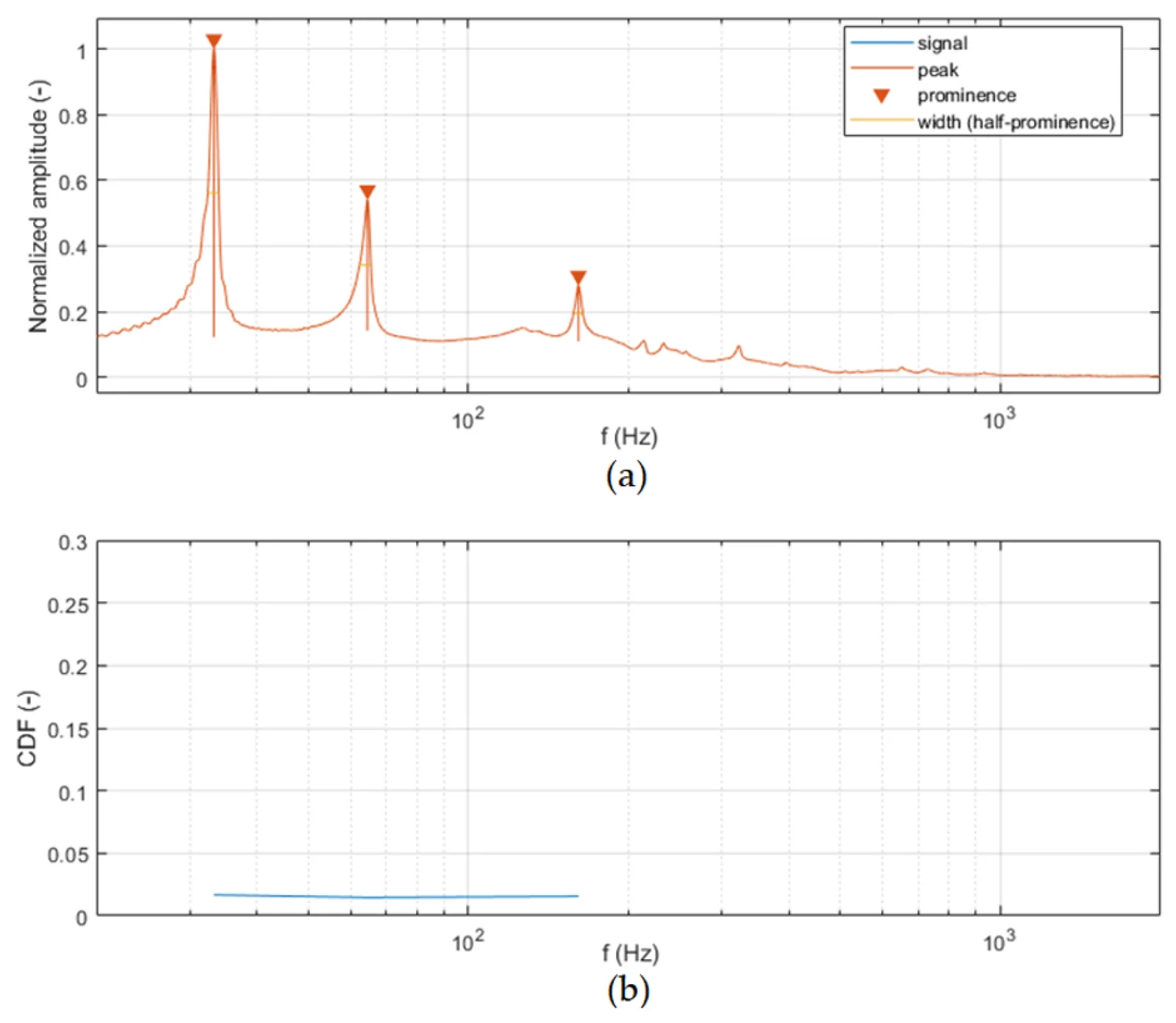
Acoustic-response analysis of a clamped EPS-T specimen under impulse excitation: (**a**) normalized single-sided amplitude spectrum with identified resonance peaks and half-prominence widths; (**b**) corresponding critical damping factors.

For each specimen, at least 10 impacts distributed over the free part of the sample were recorded simultaneously by both microphones at 96 kHz, with a record length of 1.5 s per impact. The FFT spectra from both microphones and all repeated impacts were combined into a spectral envelope by retaining the maximum amplitude at each frequency. This preserved modes visible only in one microphone channel. Resonance and half-power frequencies were identified from the combined envelope. An example of the resonance-peak identification and the corresponding damping estimates is shown in Figure 8.

Before the inverse identification procedure, a preliminary numerical modal analysis was performed for three representative specimen thicknesses: 17, 33, and 53 mm, assuming E = 1.7 MPa. This step was used to verify mode tracking and reduce the risk of ambiguous optimization caused by local minima. For the 17 mm specimen, the bending-dominated and bending–torsion modes corresponded to the first and second eigenmodes, respectively. For the 33 and 53 mm specimens, the bending–torsion mode corresponded to the third eigenmode, while the second eigenmode was a weakly radiating mode and was not consistently identifiable in the measured acoustic spectra. Therefore, the modes used for inverse identification were selected based on their deformation pattern rather than their numerical order.

**Figure 9:**
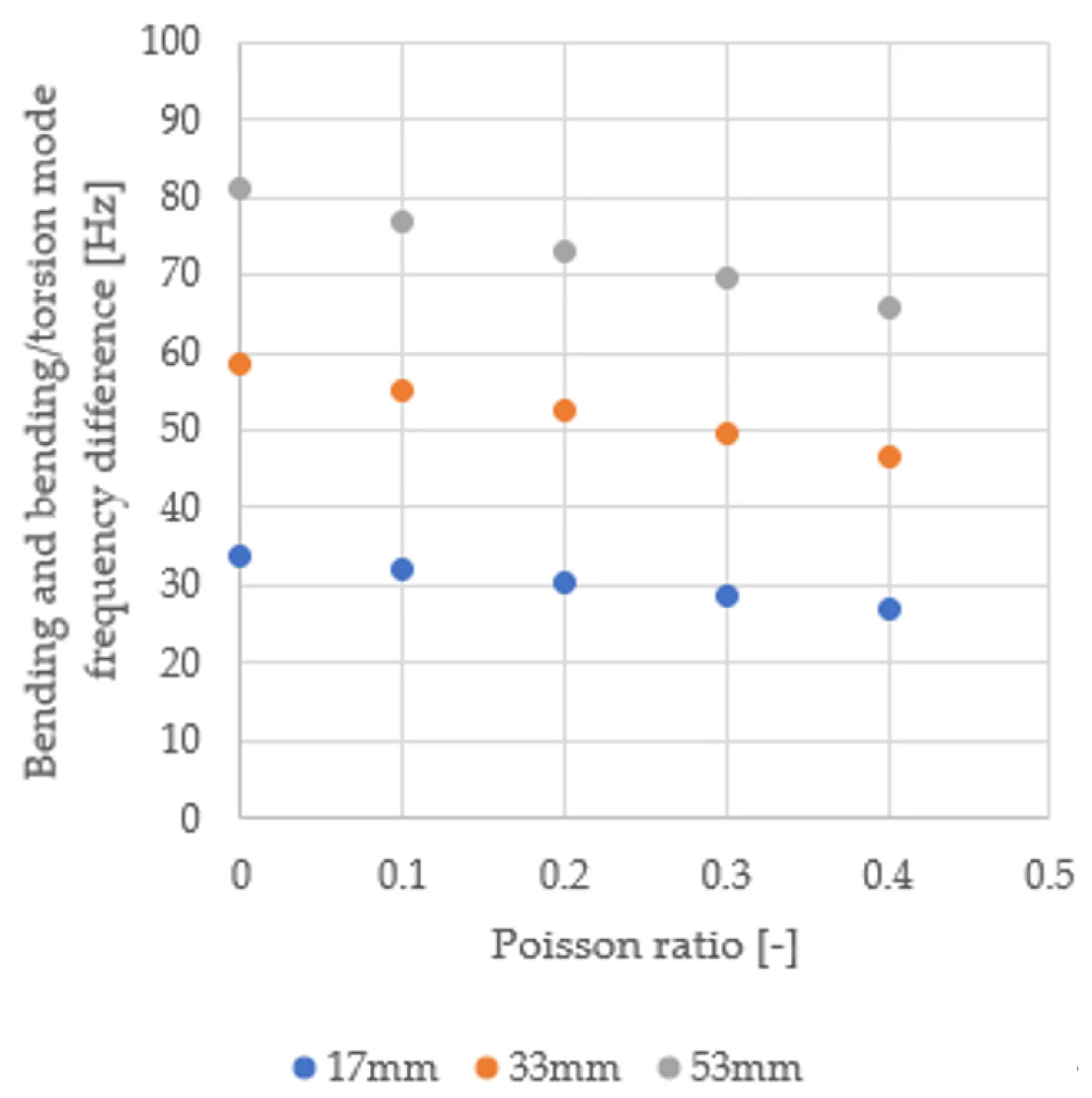
Preliminary sensitivity analysis of the frequency difference between the bending-dominated mode and the bending–torsion mode as a function of Poisson’s ratio for selected specimen thicknesses. The analysis was performed for a representative Young’s modulus of *E* = 1.7 MPa.

As shown in Figure 9, the frequency difference between the bending-dominated and bending–torsion modes decreased monotonically with increasing Poisson’s ratio for all analyzed thicknesses. No local minima were observed, reducing the risk of ambiguous inverse identification. This confirmed that the selected mode pair was suitable for simultaneous identification of Young’s modulus and Poisson’s ratio.

The experimentally identified resonance frequencies were used for inverse identification of the effective elastic parameters. A COMSOL Multiphysics v5.6 model of the clamped specimen was developed as a linear elastic isotropic solid, with E and ν treated as optimization variables. The linear elastic isotropic approximation was adopted because the inverse identification was based on low-amplitude modal response, for which material nonlinearity was expected to be limited. Viscoelastic effects were represented separately by the identified damping parameters. For low damping, the difference between the damped resonance frequency and the undamped natural frequency is small and has only a minor influence on the identification of *E* and *ν*. These parameters should therefore be interpreted as effective small-strain dynamic properties. At higher excitation amplitudes, material nonlinearity could introduce amplitude-dependent resonance frequencies and excitation-dependent equivalent parameters. The vise support was represented by fixed boundary conditions. The clamping effect was included through prestressed eigenfrequency analysis: a stationary step first introduced a 0.5 mm clamping displacement in the vise region, followed by eigenfrequency analysis around the prestressed state.

**Figure 10:**
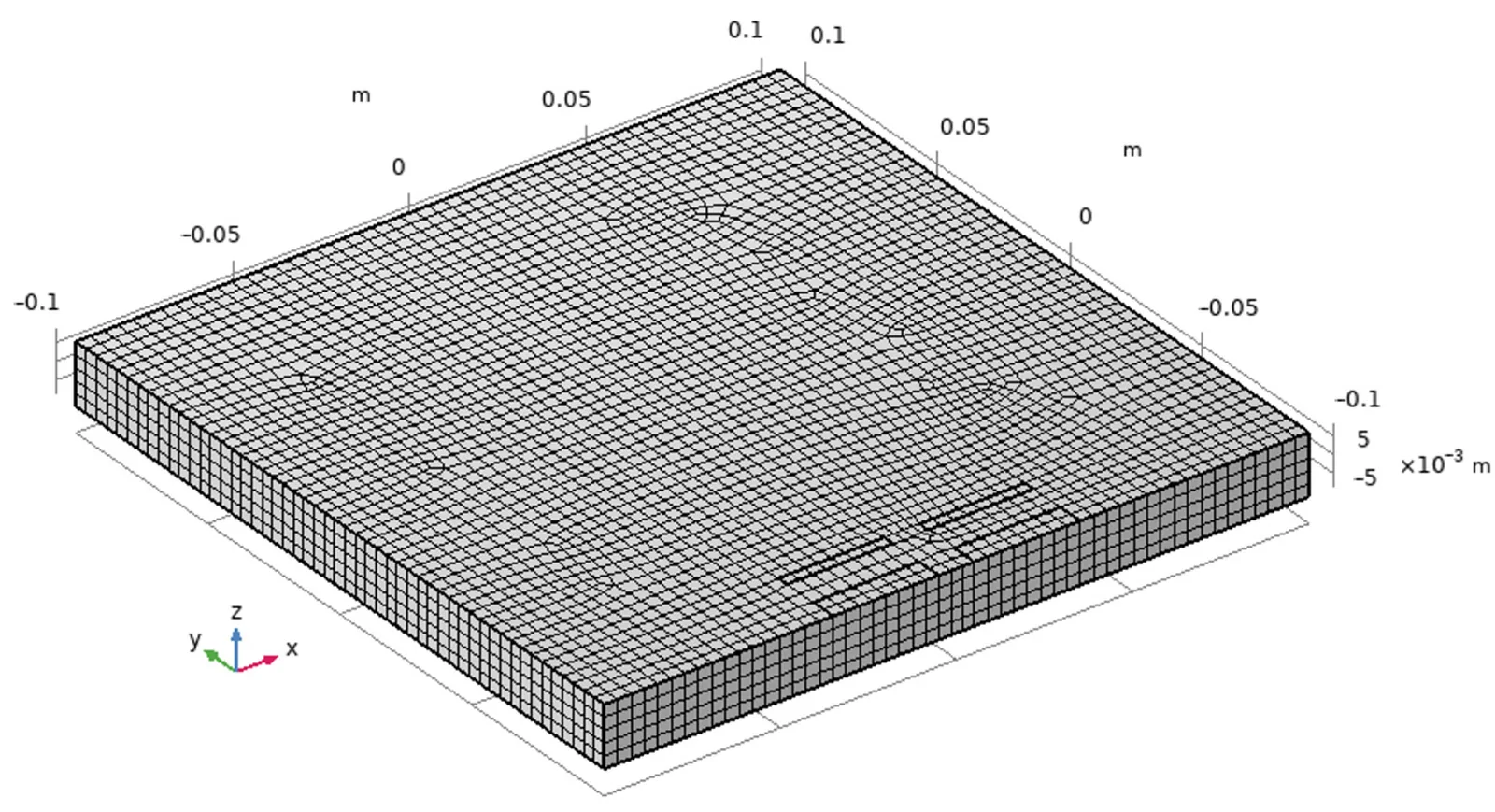
Exemplary finite-element mesh of the rectangular EPS-T specimen with thickness of 17 mm used in the modal analysis.

The FEM mesh was generated using a swept meshing procedure, producing a structured mesh composed mainly of hexahedral elements. The maximum element size was limited to 3.5 mm to provide several elements across the thickness, including the thinnest specimens. Reduced integration was used to improve computational efficiency during repeated eigenfrequency analyzes. The same meshing strategy was applied to all geometries, and an example mesh for the 17 mm specimen is shown in Figure 10.

Mesh sensitivity was evaluated for the 17 mm specimen using maximum element sizes of 7.0, 3.5, and 2.5 mm. Refinement from the adopted 3.5 mm mesh to the 2.5 mm mesh changed the identified Young’s modulus from 1.825 to 1.844 MPa and Poisson’s ratio from 0.263 to 0.265, corresponding to differences of 1.04% and 0.76%, respectively. Even between the coarsest and finest meshes, the differences were only 2.39% for Young’s modulus and 1.53% for Poisson’s ratio, indicating that the adopted mesh density had only a minor influence on the identified parameters.

The optimization problem was formulated by minimizing the discrepancy between the measured and calculated modal frequencies. For the present inverse-identification procedure, the objective function was formulated as the sum of the squared relative differences between the experimentally identified and numerically calculated modal frequencies:

(10) $$J\left(E,\nu \right)={\sum_{i=1}^{n}\left(\frac{f_{i,FEM}\left(E,\nu \right)-f_{i,exp}}{f_{i,exp}}\right)}^{2}$$

where *f_i,exp_* is the experimentally identified resonance frequency of the *i*-th mode, *f_i,FEM_* is the corresponding eigenfrequency obtained from the finite-element model, and *N* is the number of modes included in the fitting procedure. The first bending-dominated mode and the following bending–torsion mode were used as the primary fitting targets. The derivative-free Nelder–Mead algorithm was selected because the inverse problem involved only two variables and required repeated eigenfrequency calculations. Robustness was checked using a full multi-start grid with *E*_0_ = 1.5, 2.0, and 2.5 MPa and *ν*_0_ = 0.25, 0.35, and 0.45. All nine initial combinations converged to the same solution within the reported numerical precision, confirming that the identified parameters were insensitive to the initial guess within the investigated range. The optimization returned the effective Young’s modulus and Poisson’s ratio corresponding to the dynamic bending/torsional response of the material.

**Figure 11:**
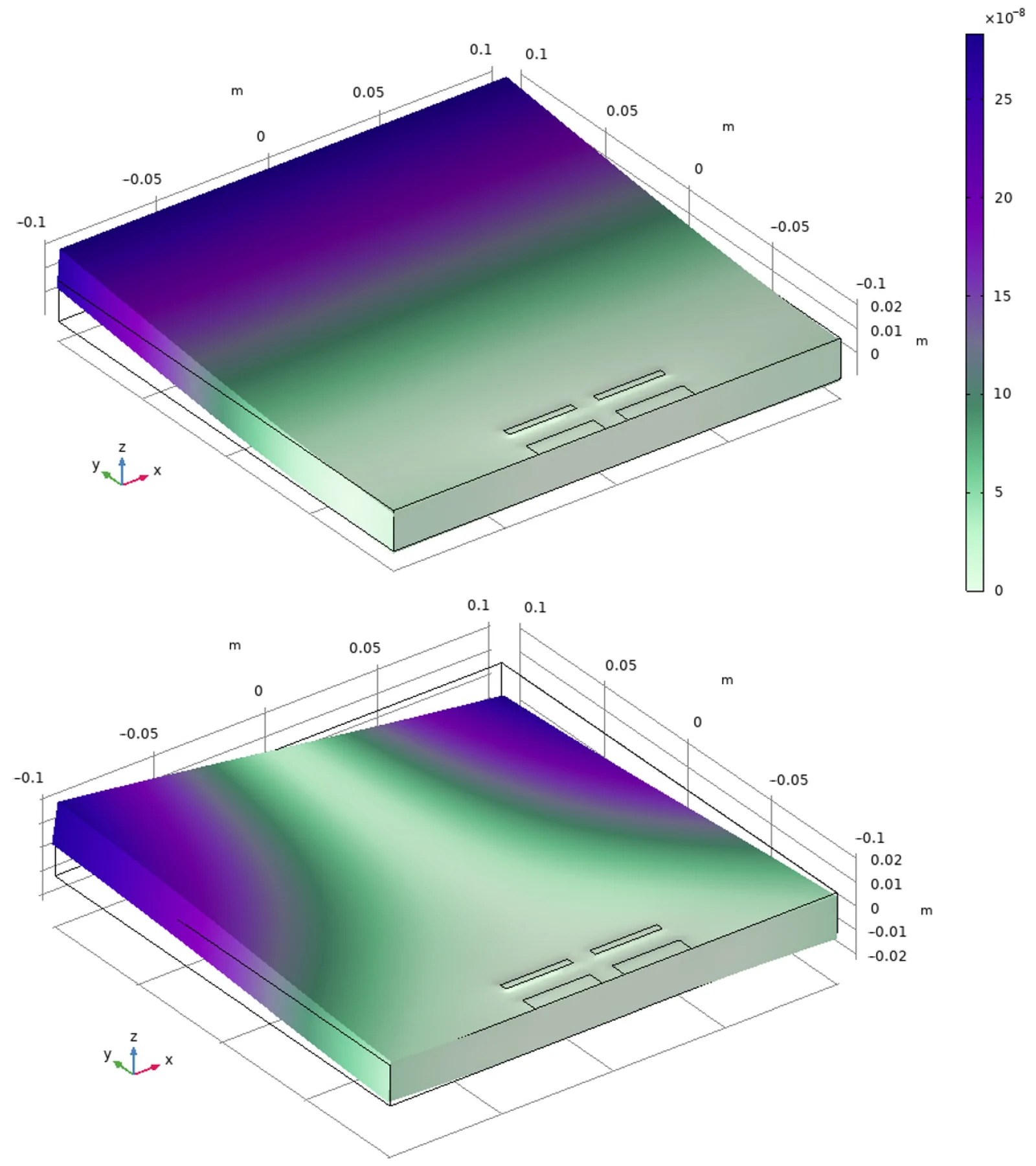
Examples of eigenmodes obtained from the optimized COMSOL model of the clamped rectangular EPS-T specimen: first bending-dominated mode and first bending–torsion mode used for inverse identification of elastic parameters. For the presented case, the optimized model yielded resonance frequencies of 33.24 Hz and 64.62 Hz, with identified material parameters of *E* = 2.07 MPa and *ν* = 0.292.

Mode selection for inverse identification was based on deformation patterns rather than eigenmode numbers. For the 17, 22, and 27 mm specimens, the bending-dominated and bending–torsion modes corresponded to the first and second eigenmodes, respectively. From 33 mm upwards, the same bending–torsion mode shifted to the third eigenmode. Therefore, modal shapes were tracked consistently across specimen thicknesses. Example mode shapes for the 17 mm specimen are shown in Figure 11.

For each nominal thickness group (*n* = 3), the results were summarized using the mean, sample standard deviation and coefficient of variation. Inferential significance testing between thickness groups was not performed because three specimens per group did not provide sufficient statistical power for reliable group comparisons.

### 2.5. FEM Consistency-Check Model of the ISO-Type Configuration

To assess the consistency of the proposed interpretation, the ISO-type test configuration was modelled using elastic parameters obtained from the acoustic-response inverse FEM procedure. This analysis was not an independent validation, because the equivalent normal contact/support stiffness was identified from the ISO-type dataset. Instead, it was used to examine whether the modal elastic parameters combined with the series-compliance interpretation could reproduce the measured resonance frequency of the ISO-type compression system.

The model used the geometry of the ISO-type dynamic stiffness test. The EPS-T layer was described by the mean effective Young’s modulus and specimen-specific Poisson’s ratios obtained from inverse modal identification. The remaining components were modelled as linear elastic isotropic materials. The contact/support contribution was introduced through the equivalent normal stiffness per unit area *s_c_*, while two tangential interface assumptions were compared: tangentially free and tangentially bonded contact. The input parameters are summarized in Table 3.

**Figure 12:**
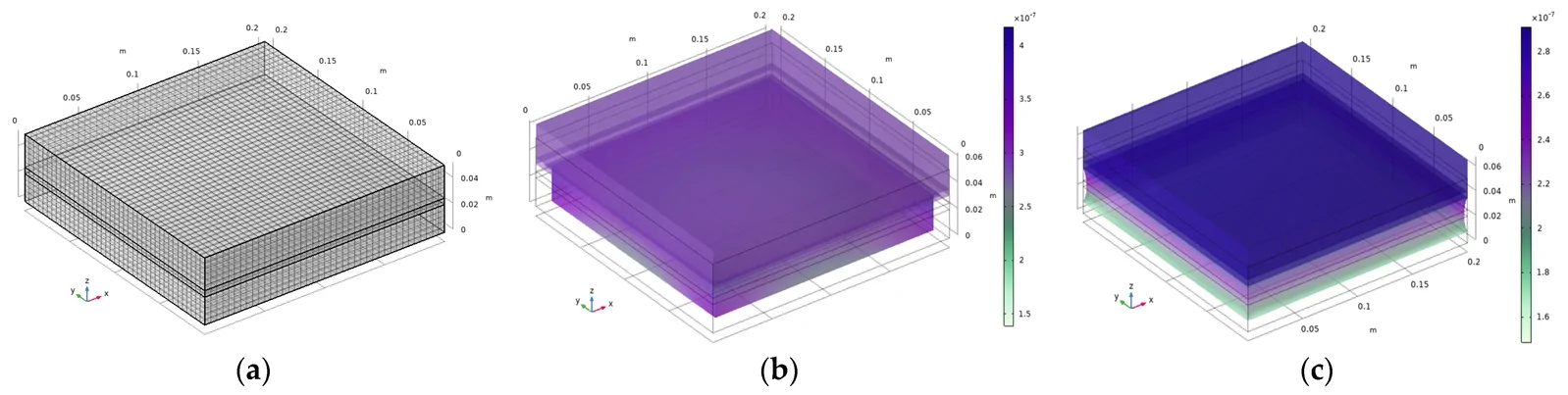
Finite-element representation of the ISO-type dynamic stiffness test configuration used in the FEM consistency check: (**a**) exemplary finite-element mesh; (**b**) first piston-like mode shape obtained for the tangentially free/slip interface assumption; (**c**) first piston-like mode shape obtained for the tangentially bonded interface assumption. Colours indicate the displacement magnitude in modal analysis.

An exemplary finite-element mesh and the corresponding first piston-like mode shapes of the ISO-type test configuration are shown in Figure 12c. For clarity, only one representative specimen geometry is presented, together with the two tangential interface assumptions considered in the FEM consistency check: tangentially free/slip contact and tangentially bonded contact.

Mesh sensitivity of the ISO-type FEM model was evaluated for the 17.05 mm specimen under the more numerically demanding tangentially bonded contact condition (Figure 12c). Maximum element sizes of 7.5, 5.0, and 3.0 mm gave first eigenfrequencies of 85.412, 85.402, and 85.396 Hz, respectively. Refinement from the adopted 5.0 mm mesh to 3.0 mm changed the eigenfrequency by only 0.007%, while the difference between the coarsest and finest meshes was 0.019%. The adopted mesh was therefore considered sufficiently converged.

## 3. Results

This section presents the experimental and numerical results obtained for the tested EPS specimens, including dynamic stiffness and damping parameters, apparent compression-based Young’s modulus, and effective elastic constants identified from the inverse FEM fitting of the measured modal response.

### 3.1. Dynamic Stiffness and Damping from ISO-Type Test

The results of the ISO-type dynamic stiffness test are summarized in Table 4, including specimen mass, unloaded thickness, critical damping factor and dynamic stiffness.

**Figure 13:**
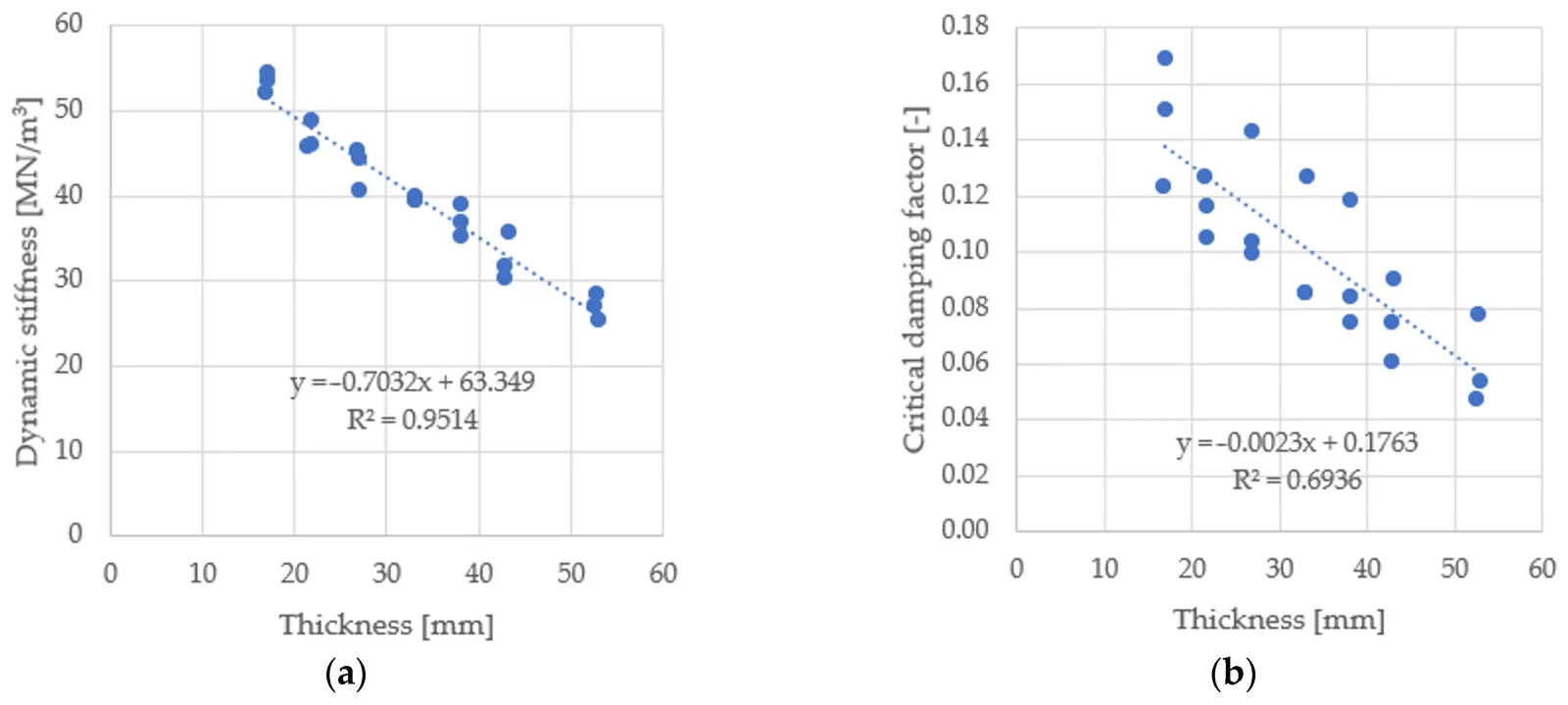
Relationship between unloaded specimen thickness and dynamic response parameters of the tested EPS specimens: (**a**) dynamic stiffness and (**b**) critical damping factor. Dashed lines indicate linear regression fits.

Dynamic stiffness decreased clearly with specimen thickness, from approximately 53.3 MN/m^3^ for the 17 mm specimens to 27.0 MN/m^3^ for the 53 mm group. The critical damping factor showed a similar but more scattered decrease, from about 0.148 to 0.059. Within the nominal thickness groups, the coefficient of variation ranged from 0.7% to 8.4% for dynamic stiffness and from 9.5% to 26.5% for the ISO-type critical damping factor. Thus, specimen thickness affected both the elastic and dissipative response of the tested EPS-T material. The results are shown in Figure 13.

Both dynamic stiffness and the critical damping factor decreased with unloaded specimen thickness. This trend was strongest for dynamic stiffness, with linear regression explaining most of the variability R^2^ = 0.9514. Damping showed the same tendency but with greater scatter between specimens of similar thickness R^2^ = 0.6936.

A second-order polynomial fit was also evaluated and increased R^2^ only marginally, from 0.9514 to 0.9570. The linear regression was therefore retained solely as a simple descriptive representation of the measured trend. The physically motivated nonlinear relationship between dynamic stiffness and thickness is analyzed separately using the series-compliance model in Section 4.1.

### 3.2. Apparent Young’s Modulus and Fitted Poisson’s Ratio Under Compression

The average geometrical parameters and resonance-based apparent Young’s modulus values obtained for the EPS-T column specimens are summarized in Table 5. These data were subsequently used to fit the effective Young’s modulus and Poisson’s ratio under compression.

The apparent Young’s modulus varied between 0.9503 MPa and 1.3979 MPa, depending on specimen height and geometrical factor. Fitting the apparent modulus–geometry relationship resulted in an effective Young’s modulus of 0.9596 MPa and a Poisson’s ratio of 0.4216, with corresponding confidence intervals of 0.8001–1.1192 MPa and 0.3322–0.5110, respectively. The coefficient was R^2^ = 0.7104, indicating a moderate agreement between the fitted model and the experimental data.

Although the fitted Poisson’s ratio was physically admissible, the upper bound of its unconstrained confidence interval slightly exceeded 0.5. This should be interpreted as limited sensitivity of the compression-based fit to *ν*, not as a physically meaningful value above the incompressibility limit. Therefore, the result indicates a relatively high effective Poisson’s ratio, with the admissible upper range bounded by *ν* < 0.5.

The confidence interval exceeding 0.5 indicates strong parameter coupling and limited identifiability of *ν* in the cylindrical compression fit, which also reduces confidence in this route as a source of final material constants. However, this uncertainty was not propagated into the subsequent FEM analyses, because the cylindrical result was used only as supporting diagnostic evidence; the FEM models used the physically admissible specimen-specific *E* and *ν* values obtained from acoustic-response inverse identification.

Consequently, the cylindrical compression fit should be regarded as supporting diagnostic evidence only. The moderate fit quality, the lower effective modulus compared with the ISO-compliance and modal FEM routes, and the limited sensitivity of the fit to Poisson’s ratio indicate that this configuration does not provide a stable basis for defining the final FEM-ready material parameters. Instead, it highlights the influence of specimen geometry, lateral constraint and contact conditions on compression-based identification.

### 3.3. Modal-Based Identification of Effective Elastic Properties

The experimentally identified sound-radiated modal frequencies were used as target values in the inverse FEM procedure. Table 6 summarizes the selected modal pairs, corresponding critical damping factors and the fitted effective Young’s modulus and Poisson’s ratio for each EPS specimen.

The first modal frequency increased with specimen thickness, from approximately 30–33 Hz for the thinnest specimens to 83–85 Hz for the thickest ones. The inverse FEM procedure gave effective Young’s modulus values of 1.77–2.16 MPa and Poisson’s ratios of 0.258–0.427. Within the nominal thickness groups, the coefficients of variation ranged from 1.2% to 8.3% for Young’s modulus and from 1.2% to 6.9% for Poisson’s ratio. The systematic variation in the identified Poisson’s ratio with specimen thickness indicates that these values should be interpreted as effective modal parameters of the clamped-specimen configuration rather than as intrinsic material constants of EPS-T.

As specimen thickness changes, the slenderness, relative extent of the clamped region, prestress distribution and sensitivity of the selected modal pair to Poisson’s ratio also change. Within the simplified linear isotropic model, the fitted *ν* may therefore partly compensate for configuration effects not represented explicitly, particularly local clamping and three-dimensional deformation near the support. The observed trend should consequently not be interpreted as evidence of an intrinsic thickness dependence of Poisson’s ratio.

## 4. Discussion

The discussion focuses on linking the three identification routes into a consistent interpretation of EPS-T vibroacoustic parameters. The key issue is not only the agreement between the identified elastic properties, but also the correct conversion between effective Young’s modulus and ISO-type dynamic stiffness, including additional compliance and configuration-dependent damping.

### 4.1. Equivalent Contact/Support Stiffness Evaluation from ISO-Type Dynamic Stiffness Data

The ISO dynamic stiffness test is commonly interpreted using a single-degree-of-freedom model, where the measured resonance frequency is converted into the dynamic stiffness of the tested layer. For soft and highly deformable materials such as EPS-T, however, the measured response may also include additional compliance from specimen surfaces, local contact conditions, the separating foil, and the support configuration.

Therefore, the measured stiffness was interpreted as the effective stiffness of a series system composed of the EPS-T layer stiffness and an equivalent contact/support stiffness. Under the same normal force, the total measured displacement is the sum of the EPS-T layer deformation and the contact/support deformation; consequently, the corresponding compliances are additive. This approach separates the thickness-dependent material contribution from the thickness-independent contribution associated with the test configuration. The latter is treated not as an intrinsic material constant, but as an equivalent normal stiffness representative of the tested specimen family and measurement procedure.

**Figure 14:**
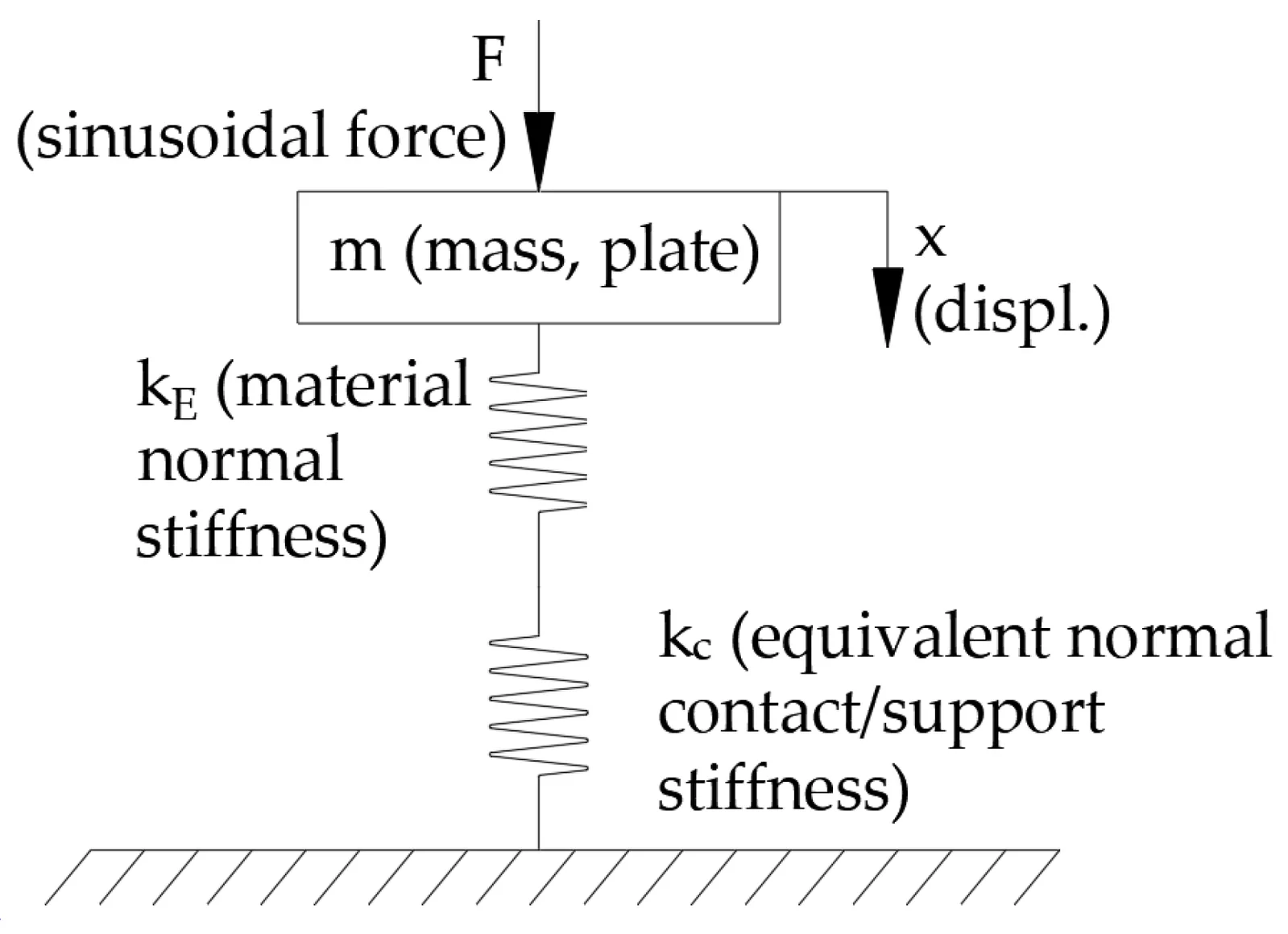
Simplified mechanical model used to interpret the ISO-type dynamic stiffness test. The measured effective stiffness is represented by two springs connected in series: the material normal stiffness of the EPS-T layer, *k_E_*, and the equivalent normal contact/support stiffness, *k_c_*.

The corresponding mechanical model is shown in Figure 14, where the measured effective stiffness is represented by two serially connected normal stiffness components: the material stiffness of the EPS-T layer, (*k_E_*), and the equivalent normal contact/support stiffness, (*k_c_*).

The proposed model can be expressed mathematically by first determining the measured effective stiffness from the resonance frequency and then representing this stiffness as two serially connected normal stiffness components (Equations (11)–(16)).

(11) $$k_{eff}=m{\left(2\pi f_{r}\right)}^{2}$$

(12) $$\frac{1}{k_{eff}}=\frac{1}{k_{E}}+\frac{1}{k_{c}}$$

(13) $$k_{E}=\frac{EA}{h}$$

(14) $$s_{c}=\frac{k_{c}}{A}$$

(15) $$s^{\prime }=\frac{k_{eff}}{A}$$

(16) $$\frac{1}{s^{\prime }}=\frac{h}{E}+\frac{1}{s_{c}}$$

This leads to a linear relationship between 1/*s*′ and specimen thickness *h*, where the slope is 1/*E* and the intercept is 1/*s_c_* or equivalently *c*_0_.

**Figure 15:**
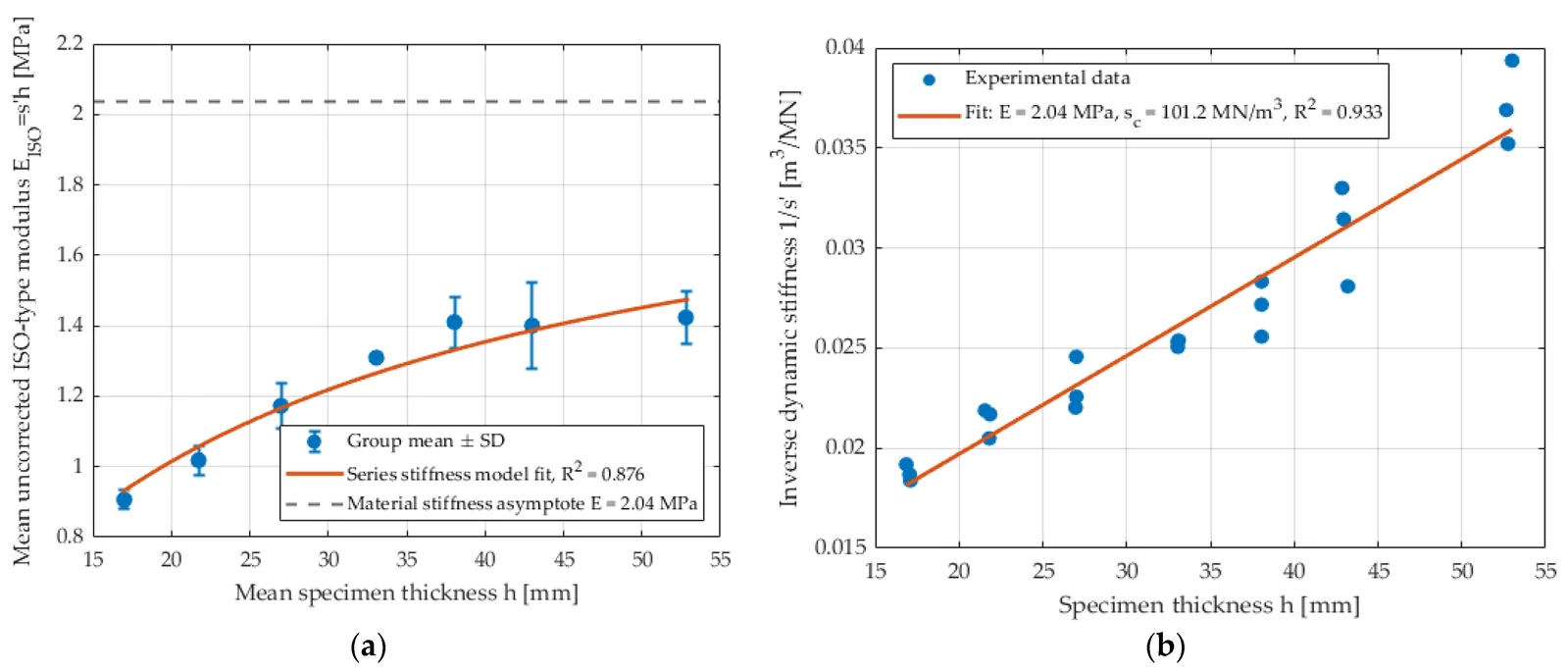
Series stiffness interpretation of ISO dynamic stiffness data: (**a**) uncorrected ISO-type modulus (*E_ISO_* = *s*′*h*); (**b**) linearized model (Equation (16)), from which *E* and *s_c_* were estimated.

Figure 15 illustrates the effect of the equivalent contact/support stiffness, the ISO-type dynamic stiffness results were first converted into the uncorrected modulus *E_ISO_* = *s*′*h*. This direct conversion produced a thickness dependent apparent modulus, indicating that the measured stiffness cannot be attributed solely to the material stiffness of the EPS-T layer. Therefore, the same data were also presented in the linearized form 1/*s*′ versus specimen thickness, which enables the material stiffness and the equivalent normal contact/support stiffness to be separated.

The linearized fit agreed well with the series stiffness model R^2^ = 0.933, indicating that the thickness dependence of the ISO dynamic stiffness can be explained by the combined effect of EPS-T layer stiffness and additional contact/support compliance. The fitted value *E* = 2.04 MPa may therefore be interpreted as an effective asymptotic modulus under normal dynamic compression, obtained after separating the thickness-independent compliance from the measured response.

The equivalent contact/support stiffness was *s_c_* = 101.2 MN/m^3^. Although higher than the measured specimen stiffness, it cannot be neglected, especially for thinner specimens where *E*/*h* becomes comparable to *s_c_*. As a result, the direct conversion *E_ISO_* = *s*′*h* underestimates the effective modulus and produces an apparent thickness dependence, as shown in Figure 15a. Thus, *s_c_* should be treated as an equivalent parameter of the tested specimen family and measurement configuration, not as a universal EPS-T material property.

To assess whether the fitted parameters were dominated by any single thickness group, a leave-one-thickness-group-out sensitivity analysis was performed. The model was successively refitted after excluding all three specimens representing each nominal thickness. The resulting parameters ranged from *E* = 1.97 to 2.33 MPa and from (*s_c_* = 86.6) to 108.4 MN/m^3^. The mean absolute percentage error for predicting the omitted dynamic-stiffness results ranged from 3.9% to 8.8%. The largest parameter shift and prediction error occurred when the nominal 53 mm group was excluded, indicating that the thickest specimens contributed appreciably to separating material and thickness-independent compliance. Nevertheless, no individual group caused a breakdown of the model. This analysis constitutes an internal robustness check rather than independent experimental validation, and the fitted *s_c_* should therefore be verified using other production batches and test configurations.

The close agreement with the mean modulus of 2.05 MPa obtained from acoustic-response inverse FEM supports the consistency of the effective modulus identified by the compliance model. However, it does not independently validate *s_c_* itself, because this parameter was fitted from the ISO-type dataset.

### 4.2. Limited Identifiability of Contact Effects in Cylindrical Compression Tests

Since the ISO-type results indicated an additional thickness-independent compliance, the same concept was tested for the cylindrical specimens. By analogy with the compliance model used in Section 4.1, an additional compliance term was introduced into the compression-based cylindrical model.

The apparent modulus was first described using the Williams–Gamonpilas relationship [53] reproduced in simplified form as Equation 17 (simplified Equation (9)).

(17) $$E_{a}=E\Phi (S,\nu )$$

where *E_a_* is the apparent Young’s modulus, E is the effective Young’s modulus, ν is Poisson’s ratio, and *Φ*(*S*,*ν*) is the shape correction factor. The aspect ratio was defined in Equation (8).

The contact-compliance extension was written in Equation (18).

(18) $$\frac{h}{E_{a}}=\frac{h}{EG(S,\nu )}+c_{cyl}$$

where *c_cyl_* is an equivalent compliance term for the cylindrical-specimen test configuration. This term should not be interpreted as a material constant, but as a parameter representing possible contact, specimen preparation, and test system effects.

Four model variants were compared:

- Model A—pure Williams–Gamonpilas [53] correction (no *c_cyl_* compliance);
- Model B—mean Poisson’s ratio from acoustic-response modal identification (*ν* = 0.361) and additional cylindrical compliance, with fitted E and *c_cyl_*;
- Model C—fitted Poisson’s ratio and additional cylindrical compliance, with fitted *E*, *ν*, and *c_cyl_*;
- Model D—thickness-specific Poisson’s ratios from acoustic-response modal identification and additional cylindrical compliance, with fitted *E* and *c_cyl_*.

**Figure 16:**
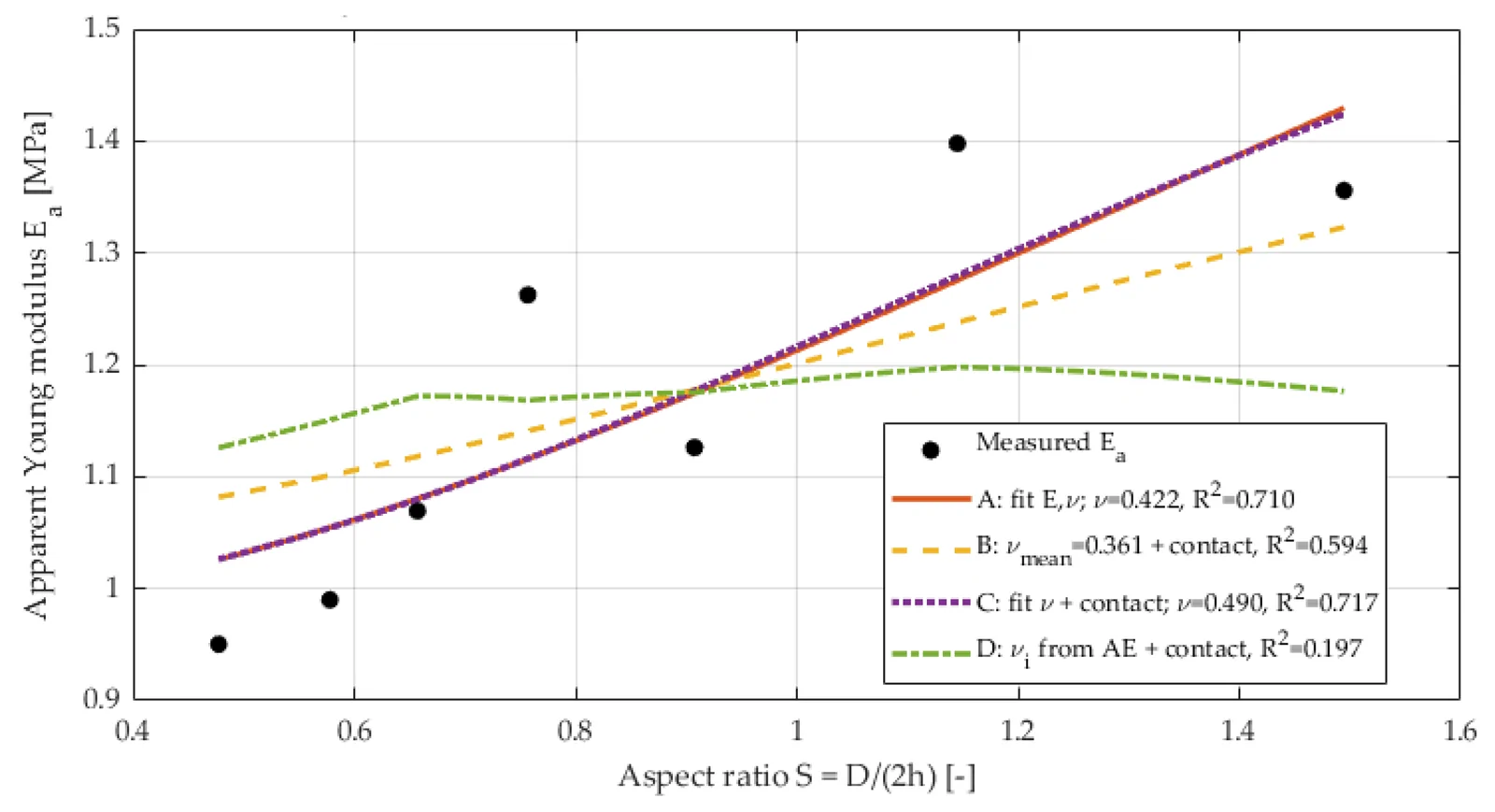
Apparent Young’s modulus of cylindrical EPS-T specimens as a function of aspect ratio (*S* = *r*/*h*). Model A represents the Kirchhoff/Williams–Gamonpilas fit, while Models B–D include an additional cylindrical compliance term (*c_cyl_*) with different assumptions for Poisson’s ratio.

The fitting results are shown in Figure 16. Model A gave *E* = 0.960 MPa, *ν* = 0.422, and R^2^ = 0.710. Model B gave *E* = 1.036 MPa, *c_cyl_* = 2.22 × 10^−14^ m^3^/MN, and R^2^ = 0.594. Model C gave *E* = 0.990 MPa, *ν* = 0.490, *c_cyl_* = 3.28 × 10^−3^ m^3^/MN, and R^2^ = 0.717. Model D gave *E* = 1.056 MPa, *c_cyl_* = 2.22 × 10^−14^ m^3^/MN, and R^2^ = 0.197.

These results show that the analogy with the rectangular ISO-type specimens did not hold for the cylindrical tests. In Models B and D, the additional compliance term became numerically negligible, while in Model C the slight improvement in R^2^ was obtained only by shifting Poisson’s ratio to the upper admissible bound. This indicates that the cylindrical compression data do not allow stable separation of material stiffness, Poisson’s ratio, aspect-ratio effects, and contact-related compliance.

This is most likely related to the higher sensitivity of the cylindrical setup to specimen preparation and boundary conditions. Possible sources include non-parallel specimen faces, cutting-induced imperfections, local bead crushing, variable friction at the loading plates, imperfect load sharing between the four cylinders, and the limited representativeness of small specimens with respect to the EPS-T bead structure.

Therefore, the lower modulus obtained from the cylindrical compression tests should be interpreted as an apparent configuration-dependent value rather than as the final effective modulus of EPS-T. Owing to strong parameter coupling and the sensitivity to specimen preparation and boundary conditions described above, this route did not provide a stable separation of material stiffness, Poisson’s ratio and contact-related effects. In contrast, the ISO-compliance fit used the full thickness series to separate the thickness-dependent material contribution from the additional compliance, while the acoustic-response inverse FEM provided a closely consistent modulus through a complementary modal-identification route. Accordingly, the common value obtained from these two routes was adopted as *E_eff_*, whereas the cylindrical result was retained only as supporting diagnostic evidence.

### 4.3. FEM Consistency Check Between the ISO-Type Test Configuration and Acoustic Modal Identification

**Figure 17:**
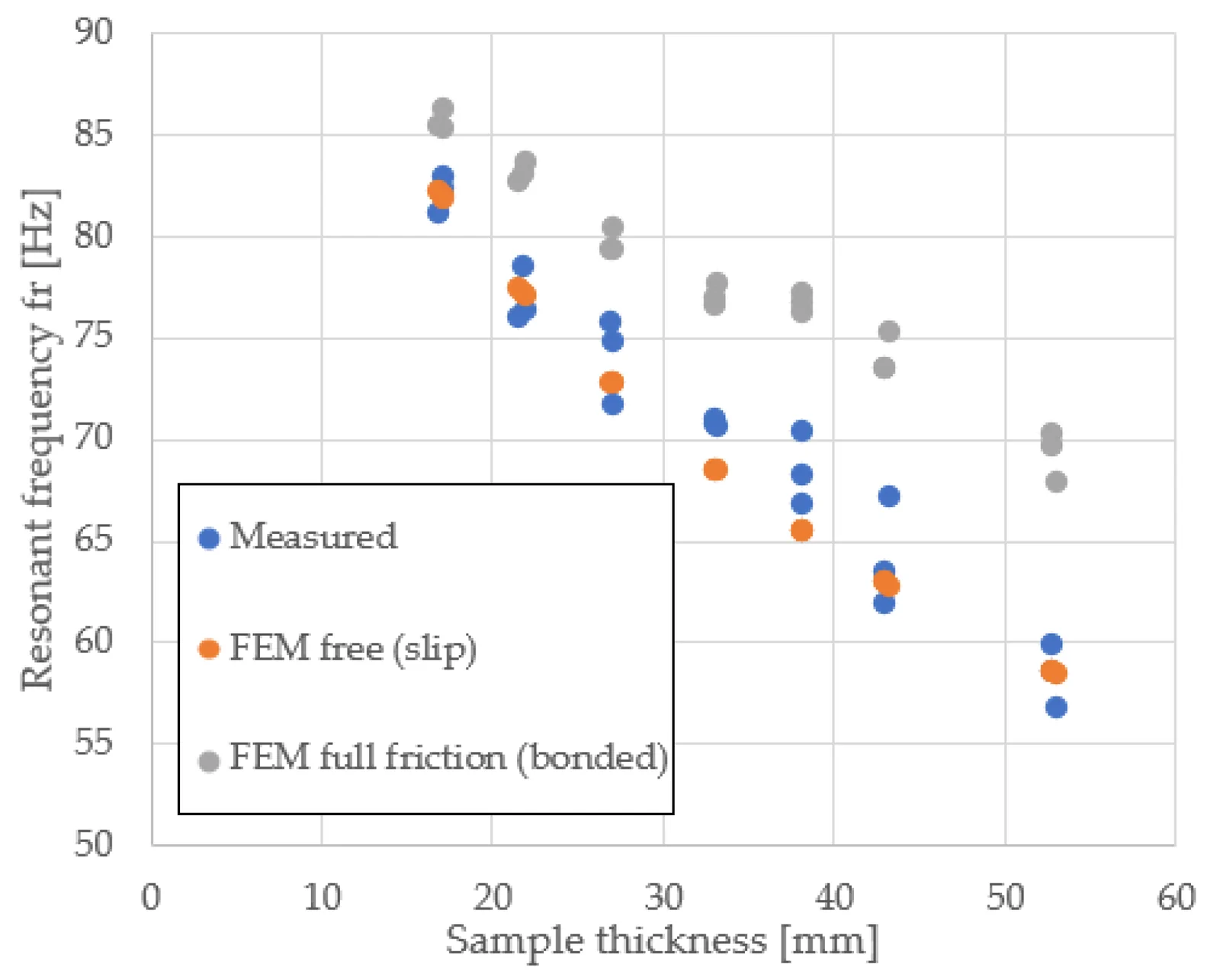
Comparison of measured ISO-type resonance frequencies and FEM predictions obtained for two tangential interface assumptions for individual specimens.

The FEM consistency check results are presented in Figure 17 while detailed group-mean resonance frequencies and relative errors are provided in Supplementary Table S1. This comparison evaluates the influence of tangential interface assumptions over the full investigated thickness range.

The results clearly indicate that the measured ISO-type resonance is better represented when tangential slip is allowed at the plate–EPS-T interface. Although both FEM variants include normal compression of the specimen, the bonded interface introduces an additional in-plane constraint, which increases the predicted stiffness and shifts the resonance frequency upward. Therefore, the discrepancy between the two numerical variants is mainly attributed to the assumed tangential boundary condition rather than to the normal support stiffness itself.

The grouped comparison confirms the specimen-level trend. With tangential slip, the mean relative error remained between approximately −4.3% and +0.4% across the investigated thickness range. In contrast, the tangentially bonded model overestimated the resonance frequency for all groups, with errors reaching +18.6% for the thickest specimens. This shows that full tangential bonding is not a realistic representation of the ISO-type test configuration.

Overall, the FEM consistency check supports the compatibility between acoustic modal identification and the ISO-type dynamic stiffness interpretation. The modal elastic parameters, combined with the equivalent normal contact/support stiffness from the series model, reproduced the measured piston-like resonance when tangential slip was allowed.

### 4.4. Engineering Consequence for EN ISO 12354-2 Impact Sound Prediction

The previous sections show that the modal Young’s modulus from inverse FEM is consistent with the asymptotic stiffness obtained from ISO-type dynamic stiffness data. However, this does not imply that it can be directly converted to the EN ISO 12354-2 floating-floor input using *s*′ = *E*/*h*. This section quantifies the practical effect of such a conversion.

For cement or calcium-sulfate floating screeds, the weighted reduction in impact sound level can be estimated from the dynamic stiffness of the resilient layer and the surface mass of the floating screed (Equation (19)):

(19) $$∆L_{w}\;=13\log_{10}m_{screed}^{\prime }\;-14.2\log_{10}s^{\prime }\;+\;20.8$$

where *m*′_screed_ is the surface mass of the floating screed in [kg/m^2^], and (*s*′) is the dynamic stiffness of the resilient layer in [MN/m^3^].

**Figure 18:**
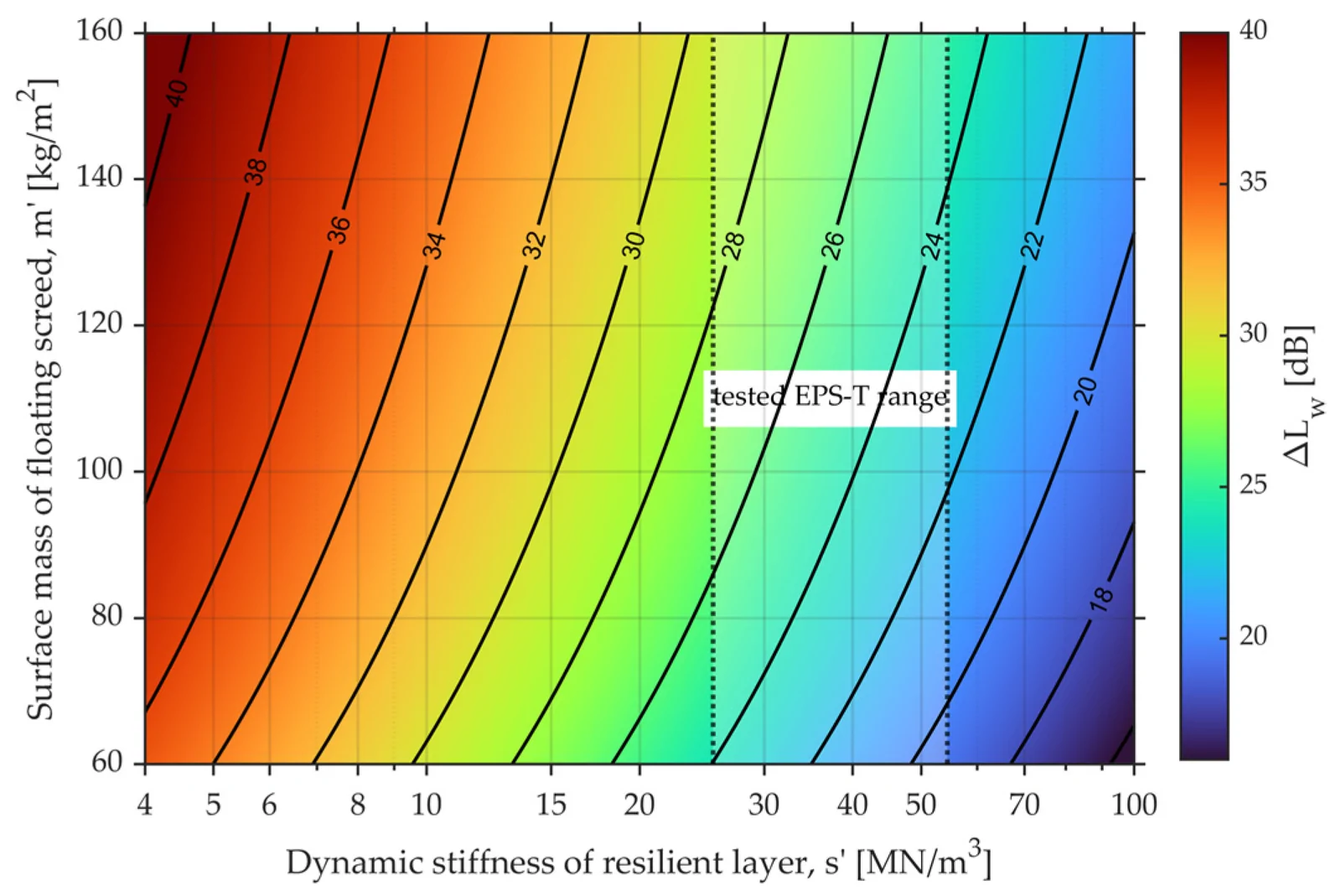
EN ISO 12354-2 estimate of weighted impact sound reduction as a function of screed surface mass and dynamic stiffness of the resilient layer.

Figure 18 presents this relationship as an engineering map for typical screed surface masses and dynamic stiffness values. The vertical band indicates the ISO-type dynamic stiffness range measured for the tested EPS-T specimens. Within this model, higher resilient-layer stiffness reduces the predicted impact sound improvement; therefore, overestimating (*s*′) directly underestimates Δ*L_w_*.

To examine this effect, three routes for defining the dynamic stiffness were compared. The first route used the directly measured ISO-type dynamic stiffness *s*′*_ISO_*, which represents the appropriate input for the standard calculation. The second route used the approach based on the Young’s modulus obtained from modal identification using Equation (20).

(20) $$s_{E/h}^{\prime }=\frac{E_{modal,mean}}{h}\;$$

where *E_modal,mean_* was obtained from the acoustic-response inverse identification as a mean value and h is the specimen thickness.

The third route incorporated the equivalent contact/support stiffness obtained from the ISO-type fit by combining the layer and contact/support compliances in series, as expressed in Equation (21), which was derived from Equation (16).

(21) $$s_{corr}^{\prime }=\frac{1}{\frac{h}{E_{modal,mean}}+\frac{1}{s_{c}}}$$

**Figure 19:**
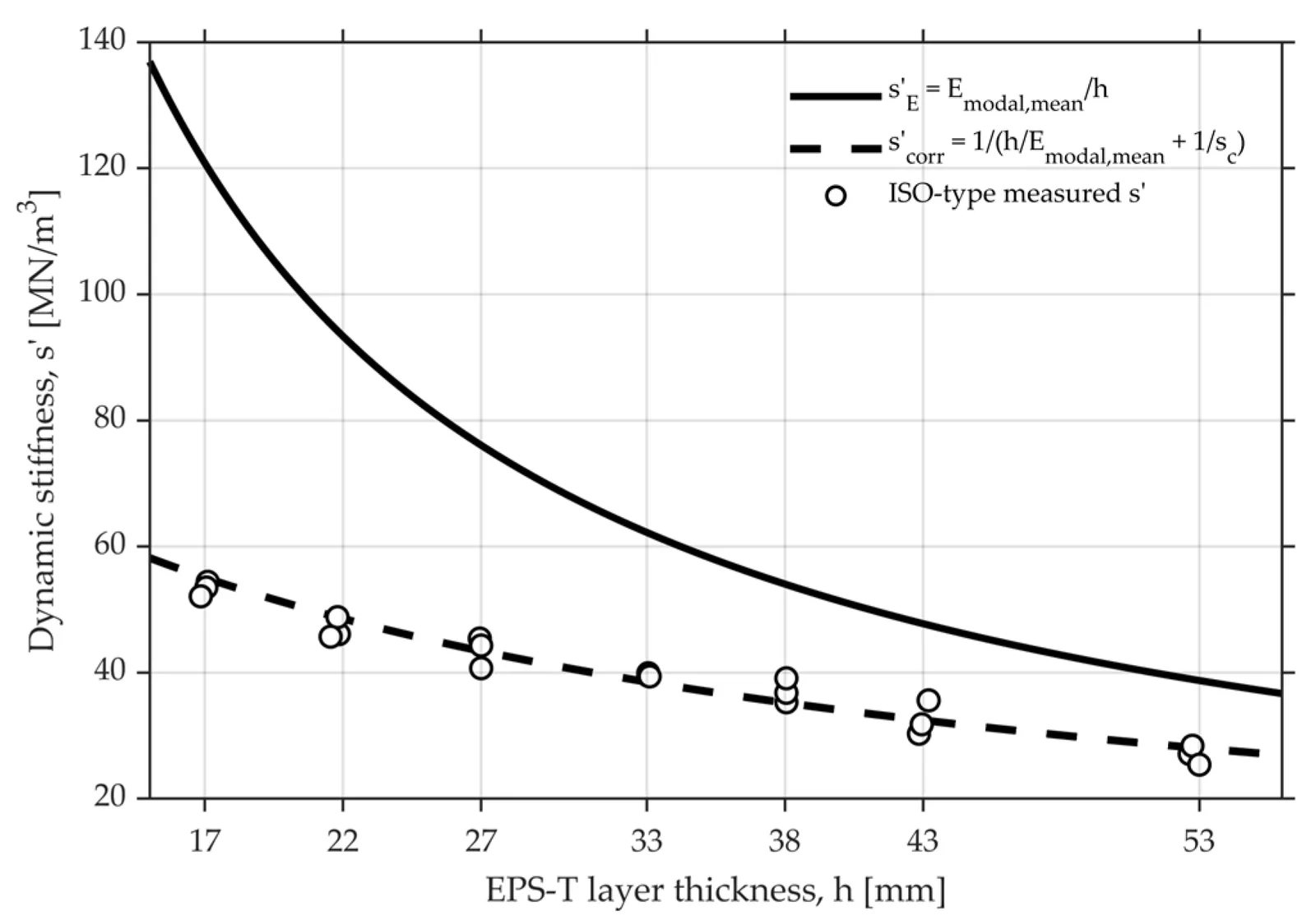
Comparison of ISO-type dynamic stiffness with stiffness estimated from modal Young’s modulus using the material-only route and the compliance-corrected route.

The comparison of these three approaches is shown in Figure 19.

Poisson’s ratio was not included explicitly in Equations (20) and (21), because these relations represent a lumped one-dimensional normal-spring conversion rather than a full three-dimensional constitutive model. In a finite specimen, the influence of Poisson’s ratio on the apparent normal stiffness depends on the degree of lateral restraint imposed by interface friction or bonding, as well as on specimen geometry and boundary conditions. Therefore, no unique correction based on ν alone can be introduced without specifying the corresponding contact conditions. In the three-dimensional FEM consistency check, Poisson’s ratio was included explicitly, and the slip-allowing interface reproduced the measured ISO-type resonance much better than the bonded interface. This indicates that a fully bonded, laterally restrained idealization is not appropriate for the tested configuration. Accordingly, Equations (20) and (21) are used only as simplified engineering conversions for comparison with the EN ISO 12354-2 input parameter. Configurations with significant lateral restraint should be analyzed using a three-dimensional model including both ν and the actual interface conditions.

The direct conversion *E_modal,mean_*/*h* gives substantially higher stiffness values than the ISO-type measurements, especially for thinner specimens. In contrast, the compliance-corrected route follows the ISO-type stiffness data much more closely. This supports the consistency of the modal Young’s modulus with the ISO-type test results within the adopted simplified normal-spring conversion, in which Poisson’s ratio was not introduced as a separate correction.

Because the surface mass term is assumed to be identical for both compared stiffness inputs, the error in predicted Δ*L_w_* caused by using *s*′*_E/h_* instead of *s*′*_ISO_* is independent of the screed mass and can be written using Equation (22).

(22) $$∆L_{w,ISO}-∆L_{w,E_{modal,mean}/h}=14.2\log_{10}\frac{s_{E}^{\prime }}{s_{ISO}^{\prime }}$$

**Figure 20:**
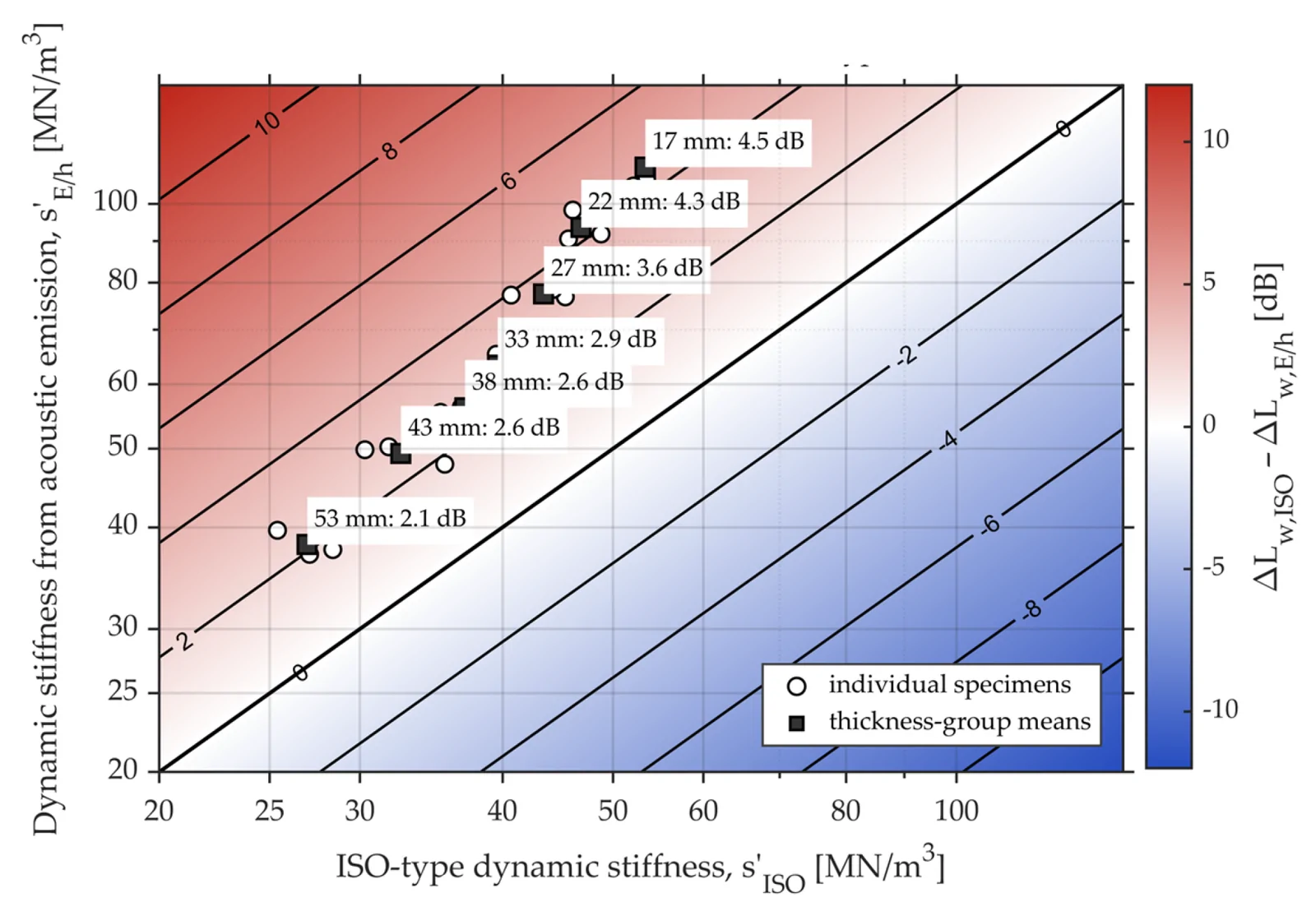
Error in the EN ISO 12354-2 predicted weighted impact sound reduction caused by using the material-only stiffness conversion *s*′*_E_*/*_h_* = *E_modal,mean_*/*h* instead of the measured ISO-type dynamic stiffness *s*′*_ISO_*. Open circles represent individual specimens, while square markers indicate mean values for the nominal thickness groups (*n* = 3). The scatter of the individual points shows the specimen-to-specimen variability in both plotted quantities. Positive contour values indicate underestimation of Δ*L_w_*.

This error is visualized in Figure 20, where the horizontal axis represents the measured ISO-type dynamic stiffness and the vertical axis represents the material-only stiffness obtained from *E*_modal,mean_/*h*. Individual markers show the results for all tested specimens, while the superimposed square markers indicate the mean coordinates for each nominal thickness group (*n* = 3). Specimen-to-specimen variability is therefore shown directly by the scatter of the individual points rather than by separate error bars.

Positive values indicate that this approach underestimates the predicted impact sound reduction. The experimental points show that the error is largest for the thinnest EPS-T specimens and decreases with increasing thickness. For the tested specimen groups, the mean error decreased from approximately 4.5 dB for the 17 mm specimens to approximately 2.1 dB for the 53 mm specimens.

This result has direct practical implications. The Young’s modulus obtained from acoustic-response modal identification is useful as a FEM-ready elastic parameter. However, using the same modulus to generate the EN ISO 12354-2 dynamic stiffness input through *E*/*h* systematically underestimates the predicted impact sound improvement. In the present case, this gives conservative results, but the error is not negligible and reaches several decibels for thin layers. Therefore, ISO-type dynamic stiffness should remain the preferred input for standard impact sound calculations, while modal elastic parameters should be used directly in FEM models or converted to *s*′ only with the additional compliance term included.

This recommendation is limited to standard EN ISO 12354-2 Annex C calculations for conventional floating-floor systems in which the resilient layer can be represented by dynamic stiffness measured under the corresponding ISO 9052-1 conditions. It should not be generalized to detailed FEM analyses, strongly nonlinear or load-dependent material behaviour, discontinuous resilient layers, non-standard boundary or contact conditions, or systems for which directly measured impact-sound reduction data are available. In such cases, parameters and modelling assumptions should be selected for the actual floor configuration rather than transferred directly from the ISO-type test.

The compliance-corrected route should not be treated as an independent validation of the ISO-type data, because *s_c_* was identified from the same ISO-type measurement set. Instead, it provides a physically interpretable link between modal elastic parameters and the normative dynamic stiffness input. Thus, *s_c_* should be regarded as an equivalent support/contact stiffness for the tested specimen family and test configuration, not as a universal material constant.

### 4.5. Effective Damping Identified from Experimental Configurations

The damping results were treated as complementary dynamic information, not as an alternative input for the EN ISO 12354-2 impact-sound prediction procedure and not as intrinsic EPS-T material constants. In this framework, floating-floor design is based mainly on the dynamic stiffness of the resilient layer and the screed surface mass. Therefore, the damping values identified in this study were used to interpret the effective dissipative response of specific experimental configurations and to provide configuration-specific input for possible numerical modelling.

The first step was to examine whether the ISO-type critical damping factor depends on the system resonance frequency. Since resonance frequency is directly related to dynamic stiffness, this comparison was used to assess whether dissipative behaviour follows the stiffness-related response of the specimen in the test bench.

**Figure 21:**
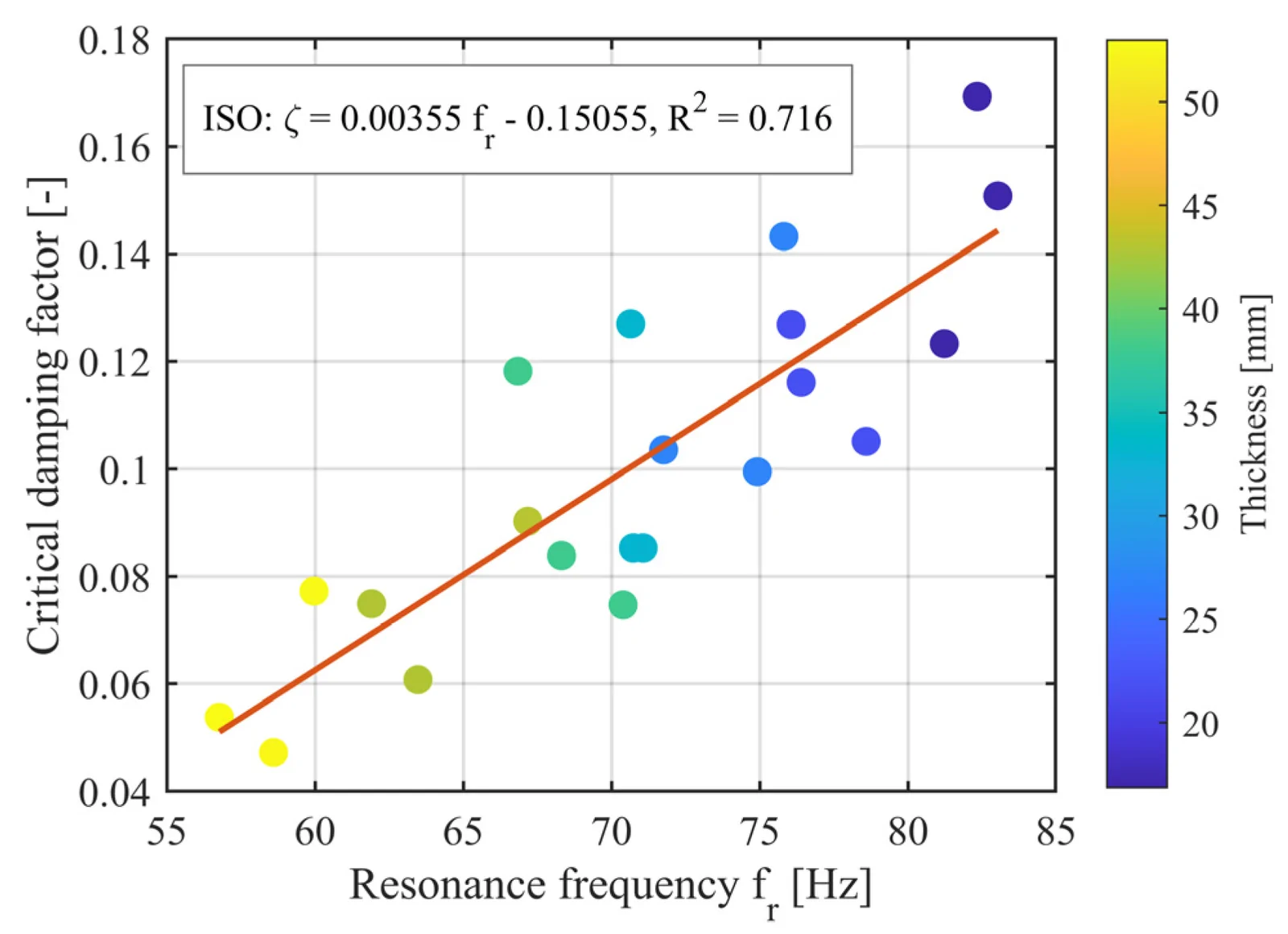
Relationship between resonance frequency and critical damping factor obtained from the ISO-type dynamic stiffness test. The colour scale denotes specimen thickness, and the solid line represents the linear regression fit.

The results presented in Figure 21 show that the ISO-type critical damping factor increases with system resonance frequency. Since resonance frequency is directly related to dynamic stiffness, the identified damping also follows the stiffness-related response of the specimen–test-bench system. This relationship was described by a linear fit with R^2^ = 0.716 shown in Equation (23).

(23) $$\zeta =0.00355f_{r}-0.15055$$

where *f_r_* is the resonance frequency in [Hz].

The damping factor obtained from the ISO-type test should not be treated as an EPS-T material damping parameter alone. It represents the response of the whole dynamic system, including the EPS-T layer, loading plate, contact interfaces, separating foil and boundary conditions. Thus, the identified damping may include both material losses and additional contact-related losses, such as local micro-slip or friction between the specimen, foil and rigid surfaces, as discussed in Section 4.3. Since these effects were not separated, the ISO-type damping factor was treated as an effective parameter of the test configuration.

Rayleigh damping coefficients were not determined for the compression-type tests. In both the ISO-type and cylindrical compression tests, each configuration provided only one dominant resonance frequency, while specimen geometry, resonance frequency and contact conditions varied between measurements. Consequently, fitting a Rayleigh-type damping model to these data did not yield stable or physically clear coefficients.

Therefore, the damping values obtained from the compression-type tests were used only to describe the effective damping of the corresponding experimental configurations. They were not used as material Rayleigh damping parameters.

Rayleigh damping [54,55] was determined only from the acoustic-response modal data. In this test, two resonance frequencies and corresponding critical damping factors were identified for each specimen, allowing the Rayleigh coefficients to be calculated using the relation in Equation (24).

(24) $$\zeta_{i}=\frac{\alpha }{4\pi f_{i}}+\beta \pi f_{i}$$

where *α* is the mass-proportional damping coefficient, and *β* is the stiffness-proportional damping coefficient. Unlike in the compression-type tests, both coefficients were obtained from two modes of the same specimen under the same boundary conditions, matching the two unknowns in the Rayleigh model. Therefore, the acoustic-response modal data provided a more consistent basis for Rayleigh damping identification.

**Figure 22:**
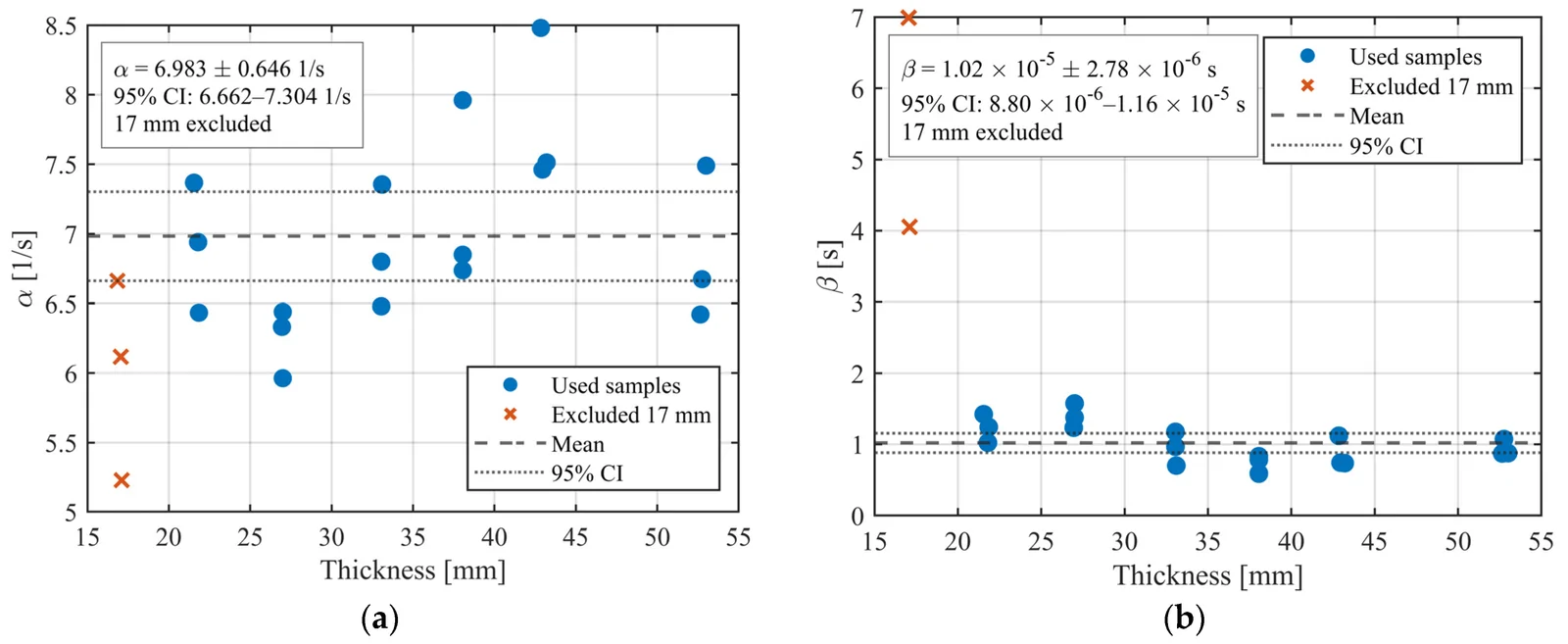
Rayleigh damping coefficients obtained from acoustic-response modal data: (**a**) mass-proportional coefficient α; (**b**) stiffness-proportional coefficient *β*. Blue markers indicate specimens used for the statistical summary, orange crosses indicate the excluded nominal 17 mm specimens, dashed lines show mean values, and dotted lines show 95% confidence intervals.

The Rayleigh damping coefficients obtained from the acoustic-response modal data are presented in Figure 22. The nominal 17 mm specimens were shown separately and excluded from the statistical summary, because their *β* values were clearly outside the range obtained for the remaining thickness groups.

The nominal 17 mm specimens were excluded from the statistical summary because their calculated β values, ranging from 4.06 × 10^−5^ to 6.99 × 10^−5^ s, were substantially higher than those obtained for the remaining specimens. This difference follows directly from the two-mode Rayleigh fit: the 17 mm specimens combined the lowest modal frequencies with comparatively high and similar damping ratios for both identified modes, which mathematically produces a larger stiffness-proportional coefficient. The thinnest specimens may also have been more sensitive to clamping conditions and to uncertainty in the half-power bandwidth identification; however, these possible experimental effects could not be separated from genuine frequency-dependent damping. The 17 mm results were therefore retained and shown separately but were not included in the pooled statistical summary. After exclusion, the mean coefficients were α = 6.983 ± 0.646 1/s with a 95% confidence interval of 6.662–7.304 1/s and *β* = 1.02 × 10^−5^ ± 2.78 × 10^−6^ s, with a 95% confidence interval of 8.80 × 10^−6^–1.16 × 10^−5^ s.

The obtained Rayleigh coefficients should therefore be regarded as effective damping parameters for FEM-type modal simulations of the tested EPS-T specimens within the investigated frequency range and under comparable clamped-boundary conditions. They should not be generalized as universal material constants or transferred directly to ISO-type compression tests, loaded floating-floor systems or models with different contact conditions. In particular, the difference between the ISO-type damping factor and the modal Rayleigh coefficients confirms that damping identification is strongly dependent on the experimental configuration, deformation mode and boundary/contact conditions.

Physically, the higher ISO-type damping includes not only material losses but also dissipation associated with compression under preload, contact interfaces, the separating foil, local micro-slip and the loading system. In contrast, the acoustic-response test describes low-amplitude bending and torsional modes of unloaded clamped specimens. The measured ranges did not overlap: ζ_ISO_ ranged from approximately 0.047 to 0.169, whereas the modal critical damping factors were approx. 0.007 to 0.024. However, no direct quantitative conversion can be established from the present data because the loading state, deformation mode, frequency and contact conditions were different.

### 4.6. Comparison with Published EPS and EPS-T Data

Directly comparable datasets reporting Young’s modulus, Poisson’s ratio and damping for elasticized EPS under similar low-amplitude conditions remain limited. Karakuş et al. directly compared elasticized and standard EPS and showed that elasticized EPS should be treated as a mechanically distinct material rather than as a softer grade of standard EPS [56]. The effective Young’s modulus obtained in the present study, 1.77–2.16 MPa, was lower than the quasi-static compressive moduli of 2.7 and 4.8 MPa reported by Chen et al. for standard EPS with densities of 13.5 and 28 kg/m^3^, respectively [57]. This difference is consistent with the lower density of the present EPS-T specimens, approximately 10 kg/m^3^, their elasticized structure, and differences in loading mode and strain range.

The modal Poisson’s ratio identified in the present study, 0.258–0.427, was higher than the values of 0.23 and 0.09 reported by Słowiński and Delalicz de Lawal for cubic and rectangular standard-EPS specimens, respectively [58]. Their strong dependence on specimen geometry supports the present interpretation of ν as an effective configuration-dependent parameter rather than a universal intrinsic constant. The modal damping ratios obtained here, approximately 0.007–0.024, were comparable in order of magnitude with the damping ratio of approximately 1% reported for EPS geofoam at cyclic strains below 0.1% [59]. In that study, damping increased towards 10% at strains above 1%, confirming its dependence on deformation amplitude. The higher ISO-type damping ratios measured in the present study, 0.047–0.169, therefore most likely include additional dissipation associated with preload, contact interfaces and the test system.

### 4.7. Configuration-Dependent Material Card for EPS-T Vibroacoustic Modelling

The parameter set proposed in Table 7 should not be treated as a universal EPS-T material card. Several quantities, especially the equivalent contact/support stiffness and damping parameters, are effective values associated with the specific test configuration, deformation mode and boundary/contact conditions. Therefore, the proposed set should be interpreted as application-oriented modelling input for comparable vibroacoustic configurations, rather than as manufacturer-type datasheet parameters.

The uncorrected conversion *s*′ = *E_eff_*/*h* is not recommended for EN ISO 12354-2 impact-sound prediction because it neglects the additional compliance and overestimates the resilient-layer stiffness. For the tested thickness groups, this led to an underestimation of Δ*L_w_* of approximately 4.5, 4.3, 3.6, 2.9, 2.6, 2.6 and 2.1 dB for nominal 17, 22, 27, 33, 38, 43 and 53 mm specimens, respectively. The compression-based Poisson’s ratio from cylindrical specimens *ν* = 0.422 should be treated only as Supporting Information due to limited identifiability and moderate fit quality.

Table 7 shows that the ISO-compliance interpretation and acoustic-response inverse FEM provide consistent estimates of *E_eff_* for the tested EPS-T family. However, this consistency does not justify direct conversion of *E_eff_* into ISO-type dynamic stiffness without the additional compliance term. The damping parameters are also configuration-specific: *ζ_ISO_* describes the loaded ISO-type compression setup, whereas the Rayleigh coefficients describe the unloaded clamped modal configuration. Thus, the proposed set should be treated as a practical bridge between ISO-type acoustic characterization and FEM modelling, not as a universal EPS-T material card.

### 4.8. Practical Limitations

The proposed identification framework is configuration dependent. Surface preparation, separating foil, contact pressure and support conditions may affect the equivalent compliance identified from the ISO-type test, while clamping and interface assumptions influence the modal parameters and damping. Moreover, the tests were performed at a nominal pressure of approximately 2 kPa, using specimens from one production batch and within a limited range of environmental conditions. Therefore, the reported parameters should not be generalized to other preparation methods, loading levels, batches or boundary conditions without additional verification.

## 5. Conclusions

### 5.1. Study Conclusions

The ISO-compliance interpretation and acoustic-response inverse FEM provided consistent estimates of the effective small-strain Young’s modulus for the tested EPS-T family, with a common value of approximately 2.04–2.05 MPa. This agreement applies to modulus identification only and does not justify the direct conversion *s*′ = *E_eff_*/*h*. The ISO-type response also contained a thickness-independent compliance equivalent to a normal contact/support stiffness of 101.2 MN/m^3^.

The cylindrical compression route did not provide equally stable material parameters. Its lower modulus and weakly identifiable Poisson’s ratio were affected by specimen geometry, contact conditions, load sharing and parameter coupling. Accordingly, this route should be treated as supporting diagnostic evidence rather than as the primary source of FEM-ready constants.

The FEM consistency check showed that the tangential interface assumption strongly affected the predicted ISO-type resonance. Allowing tangential slip reproduced the measured response much more accurately, whereas full tangential bonding systematically increased the predicted stiffness and resonance frequency. Normal compliance and tangential restraint should therefore be specified separately in numerical models.

For EN ISO 12354-2 Annex C calculations, measured ISO-type dynamic stiffness remains the preferred standardized input. Direct conversion from the identified modulus overestimated resilient-layer stiffness and underestimated the predicted impact-sound reduction by approximately 2.1 to 4.5 dB. A compliance-corrected conversion gave much closer agreement but remained specific to the tested material family and measurement configuration.

Damping parameters were also configuration dependent. ISO-type damping represented the loaded compression system, including contact and support losses, whereas the Rayleigh coefficients described the low-amplitude modal response of unloaded clamped specimens. These quantities should not be transferred directly between different test or modelling configurations.

Overall, the proposed framework provides a practical link between standardized acoustic testing and FEM-oriented modelling only when the effective modulus, contact/support compliance, tangential interface condition and damping are treated as separate configuration-dependent quantities.

### 5.2. Further Studies

Further work should first focus on separating material compliance from contact and support effects in ISO-type dynamic stiffness tests. The influence of surface preparation, separating foil, plate roughness, preload, contact pressure and repeated loading should be examined to determine whether the additional compliance term can be generalized for a given EPS-T product family or must be identified for each test configuration.

A second key direction is the controlled investigation of tangential contact at the plate–EPS-T interface. The present FEM consistency check showed that tangential bonding overestimates the ISO-type resonance frequency, whereas tangential slip gives much better agreement. Future tests should therefore compare free-slip, foil-separated, lubricated and intentionally bonded interfaces to quantify the influence of tangential restraint on dynamic stiffness and damping.

The compliance-corrected conversion from *E_eff_*  to ISO-type dynamic stiffness should also be validated on complete floating-floor assemblies. Tests with realistic screed masses, floor dimensions and boundary conditions are needed to verify whether the corrected stiffness input improves Δ*L_w_* prediction compared with the direct *E*/*h* route.

Further research should address damping transfer between configurations. ISO-type damping, modal Rayleigh damping and damping in loaded floating-floor systems should be compared directly to define when damping parameters identified in one test configuration can be safely used in another.

The apparent thickness-related trend of modal Poisson’s ratio also requires further verification. Combining modal testing with full-field deformation measurements, such as digital image correlation, and microstructural assessment of the EPS-T bead arrangement could help determine whether this trend reflects material variation, specimen-scale effects or inverse-identification artefacts.

More broadly, advanced FEM methodologies may be extended to thermo-mechanical loading and fire exposure by incorporating temperature-dependent material properties and degradation mechanisms, as demonstrated for other construction materials under standardized fire conditions [60].

Finally, the present interpretation should not be transferred directly to ETICS systems. In ETICS, discontinuous adhesive bonding, anchors, reinforcement layers and substrate interaction introduce stiffness bridges and non-uniform boundary conditions. Future ETICS-oriented studies should therefore combine the FEM-ready EPS-T parameters identified here with models including realistic adhesive patterns, anchor layouts and contact conditions.

## Figures and Tables

**Table 1:** Test bench parameters used for dynamic stiffness and damping evaluation [41].

Device Name/Manufacturer	Key Value of Parameters
Dynamic exciter—Brüel & Kjær (Virum, Denmark) Mini-shaker Type 4810	Sine peak max 10 N Frequency range DC-18 kHz
Force sensor—Forsentek (Shenzhen, China) FSSM 50 N	Max force 50 N Rated output 2.0 mV/V Hysteresis ± 0.1% R.O. (rated output)
IEPE accelerometer—MMF (Radebeul, Germany) KS78B.100	Peak acceleration 60 g (~600 m/s^2^) Linear frequency range (5% deviation) 0.6 Hz–14 kHz
Dynamic stiffness test bench	Linear frequency range upper limit (5% deviation) 20–350 Hz—measured

**Table 2:** Test bench parameters used for acoustic response evaluation.

Device Name/Manufacturer	Key Value of Parameters
GRAS 46AF free-field microphone set (Holte, Denmark)	Frequency range: 3.15 Hz–20 kHz; sensitivity: 50 mV/Pa; dynamic range: 17 dB(A)–149 dB
GRAS 12AR 2-channel power module (Holte, Denmark)	2 input/output channels; traditional 200 V microphone polarization; selectable 0/200 V polarization; preamplifier supply ±15 V; frequency response 0.05 Hz–200 kHz ±0.2 dB
RME ADI-2/4 Pro SE audio interface/AD converter (Haimhausen, Germany)	2 analog inputs/4 analog outputs; 32-bit/up to 768 kHz conversion; SNR up to 123 dBA; selectable input reference level from +1 to +24 dBu
In-house fabricated excitation hammer (Kraków, Poland)	Used for manual impulse excitation of the clamped EPS-T specimen

**Table 3:** Input parameters and modelling assumptions used in the FEM consistency check of the ISO-type dynamic stiffness test configuration.

Model Component/Assumption	Input Parameter	Value
Steel loading plate	*E*, *ν*, *ρ*	210 GPa, 0.30, 7850 kg/m^3^
Plaster of Paris layer	*E*, *ν*, *ρ*	4.6 GPa, 0.34, 1170 kg/m^3^
EPS-T specimen	*E*	2.05 MPa (mean from inverse modal identification)
EPS-T specimen	*ν*	specimen-specific, from inverse modal identification
EPS-T specimen	*ρ*	calculated from specimen mass and geometry
Equivalent normal contact/support stiffness	*s_c_*	101.2 MN/m^3^
Plate/plaster—EPS-T tangential interface condition	*-*	tangentially free/slip or tangentially bonded
Finite element mesh	maximum element size, type	5 mm, hexahedral

**Table 4:** Basic physical properties and experimentally determined dynamic parameters of the EPS specimens, including mass, unloaded thickness, critical damping factor and dynamic stiffness.

No.	Mass [g]	Thickness (No Load) [mm]	Critical Damping Factor [-]	Dynamic Stiffness [MN/m^3^]
1	7.15	17.10	0.151	54.4
2	7.05	17.05	0.169	53.5
3	6.95	16.85	0.123	52.1
4	9.06	21.85	0.116	46.1
5	8.97	21.80	0.105	48.8
6	9.25	21.55	0.127	45.7
7	10.9	26.95	0.143	45.4
8	11.25	27.00	0.099	44.3
9	11.01	27.00	0.104	40.7
10	13.76	33.05	0.085	39.5
11	13.48	33.05	0.085	39.9
12	13.31	33.10	0.127	39.4
13	15.26	38.05	0.118	35.3
14	15.39	38.05	0.084	36.8
15	15.76	38.05	0.075	39.1
16	17.26	43.20	0.090	35.6
17	17.37	42.85	0.075	30.3
18	17.76	42.95	0.061	31.8
19	21.15	52.65	0.047	27.1
20	21.14	52.75	0.077	28.4
21	21.78	53.00	0.054	25.4

**Table 5:** Average geometry, resonant frequency and apparent Young’s modulus of EPS-T column specimens used for fitting of Young’s modulus and Poisson’s ratio.

Avg. Height of Samples in Test Batch [mm]	Avg. Diameter of Samples in Test Batch [mm]	Resonant Frequency [Hz]	Apparent Young’s Modulus [MPa]	Aspect Ratio [-]	Fitting Results		
					Young’s Modulus [MPa]	Poisson’s Ratio [-]	R^2^
16.813	50.26	56.41	1.3558	1.495	0.9596 (0.8001, 1.1192)	0.4216 (0.3322, 0.5110 *)	0.7104
21.860	50.06	50.04	1.3979	1.145			
27.613	50.12	40.01	1.1261	0.908			
33.090	50.09	38.68	1.2625	0.757			
38.108	50.08	33.16	1.0695	0.657			
43.383	50.15	29.95	0.9900	0.578			
52.503	50.13	26.66	0.9503	0.477			

* Unconstrained 95% confidence interval; values above ν = 0.5 are not physically admissible for an isotropic linear elastic material and indicate parameter uncertainty.

**Table 6:** Sound-radiated modal frequencies, corresponding modal critical damping factors and inverse FEM identification results for the tested EPS-T specimens.

No.	Modes Used	Thickness [mm]	Selected Modal Frequencies [Hz]		Corresponding Modal Critical Damping Factor [-]		Young’s Modulus *E* [MPa]	Poisson’s Ratio *ν* [-]
			First	Second or Third	First	Second or Third		
1	1, 2	17.10	33.24	64.62	0.01675	0.01467	2.0656	0.2924
2	1, 2	17.05	31.13	61.18	0.02248	0.02140	1.8256	0.2630
3	1, 2	16.85	30.42	59.84	0.02376	0.02131	1.7727	0.2577
4	1, 2	21.85	42.57	81.24	0.01369	0.00947	2.1471	0.3326
5	1, 2	21.80	41.06	78.72	0.01477	0.00954	2.0018	0.3204
6	1, 2	21.55	39.18	75.49	0.01671	0.01114	1.9512	0.3082
7	1, 2	26.95	50.64	96.37	0.01191	0.00896	2.0709	0.3324
8	1, 2	27.00	50.82	95.72	0.01228	0.00949	2.1187	0.3509
9	1, 2	27.00	50.73	95.78	0.01186	0.00969	2.0850	0.3339
10	1, 3	33.05	60.37	113.61	0.01079	0.00821	2.1587	0.3610
11	1, 3	33.05	59.75	112.22	0.01083	0.00874	2.0657	0.3652
12	1, 3	33.10	60.34	112.84	0.01102	0.00766	2.0585	0.3759
13	1, 3	38.05	68.42	126.68	0.01053	0.00735	2.1080	0.4085
14	1, 3	38.05	68.56	127.10	0.00950	0.00734	2.1383	0.3988
15	1, 3	38.05	68.28	126.54	0.00977	0.00761	2.1635	0.4040
16	1, 3	43.20	74.85	137.55	0.00971	0.00752	2.0665	0.4220
17	1, 3	42.85	74.60	138.33	0.01167	0.00974	2.1354	0.4012
18	1, 3	42.95	74.39	137.55	0.00971	0.00752	2.1582	0.4023
19	1, 3	52.65	83.29	152.68	0.00841	0.00752	1.9511	0.4274
20	1, 3	52.75	83.94	154.29	0.00916	0.00864	1.9811	0.4212
21	1, 3	53.00	85.18	157.80	0.00934	0.00811	2.1024	0.4015

**Table 7:** Parameter set for EPS-T vibroacoustic modelling.

Quantity	Symbol	Value	Interpretation/Recommended Use
Effective Young’s modulus confirmed by ISO-compliance interpretation and acoustic-response modal identification	*E_eff_*	2.04–2.05 MPa	Effective small-strain dynamic modulus consistently obtained from ISO-type compliance interpretation and acoustic-response inverse FEM identification. Applies to modulus identification only.
Uncorrected stiffness estimate	*s*′*_E/h_*	*E_eff_*/*h*	Not recommended for EN ISO 12354-2 input because it neglects contact/support compliance and overestimates stiffness.
Additional thickness-independent compliance	*c_0_*	9.88 × 10^−3^ m^3^/MN	Thickness-independent compliance associated with the tested EPS-T family and ISO-type configuration; not an intrinsic material constant.
Equivalent normal contact/support stiffness	*s*_*c*_ = 1/*c*_0_	101.2 MN/m3	Normal support/contact stiffness required to reproduce the ISO-type compression response in FEM or analytical stiffness conversion.
Corrected stiffness estimate from elastic modulus	*s*′*_corr_*	(*h*/*E*_*eff*_ + *c*_0_)^−1^	Recommended conversion from E_eff_ to ISO-type dynamic stiffness when direct ISO-type s′ is unavailable.
FEM interface condition	*—*	normal compliance + tangential slip/free contact	Recommended for reproducing the ISO-type configuration; full tangential bonding overestimates resonance frequency.
Modal Poisson’s ratio from acoustic-response inverse FEM	*ν_modal_*	0.258–0.427, mean 0.361	Specimen-specific value for FEM models reproducing the unloaded clamped modal configuration. Use ν = 0.36 as a simplified representative value.
ISO-type critical damping factor	*ζ_ISO_*	Equation (23) *ζ* = 0.00355*f_r_* − 0.15055	Effective damping of the loaded ISO-type dynamic stiffness setup; not directly transferable to Rayleigh damping.
Configuration-specific Rayleigh mass-proportional damping coefficient	*α*	6.983 ± 0.646 1/s 95% CI 6.662–7.304 1/s	Effective parameter for FEM-type modal simulations of unloaded clamped specimens. Nominal 17 mm specimens excluded.
Configuration-specific Rayleigh stiffness-proportional damping coefficient	*β*	1.02 × 10^−5^ ± 2.78 × 10^−6^ s, 95% CI 8.80 × 10^−6^–1.16 × 10^−5^ s	Effective parameter for the tested acoustic-response modal configuration and frequency range; not transferable to ISO-type compression or loaded floating-floor systems.

## Data Availability

The original contributions presented in the study are included in the article, further inquiries can be directed to the corresponding author.

## Supplementary Materials

The following supporting information can be downloaded at https://www.mdpi.com/article/10.3390/ma19163540/s1. Table S1: Comparison of measured ISO-type resonance frequencies with FEM predictions obtained using tangentially free/slip and tangentially bonded interface assumptions.

## Abbreviations

The following abbreviations are used in this manuscript:

EPS-T	Elasticized expanded polystyrene
FEM	Finite element method
IEPE	Integrated electronics piezo-electric
ETICS	External thermal insulation composite system
