# Supplementary material for: Linking ISO Dynamic Stiffness and Acoustic Modal Identification for FEM-Oriented Modelling of Elasticized Expanded Polystyrene
Source: Materials (Basel). 2026 Aug 20;19(16):3540. doi: 10.3390/ma19163540 (PMC13514873; doi:10.3390/ma19163540)
Supplement: Supplementary file 1 [file materials-19-03540-s001.zip › materials-4468959-supplementary.pdf]

Supplementary Materials

Linking ISO Dynamic Stiffness and Acoustic Modal Identification for FEM-Oriented Modelling of Elasticized Expanded Polystyrene

Krzysztof Nering, Konrad Nering, Ewa Kozak-Jagieła

Table S1. Comparison of measured ISO-type resonance frequencies with FEM predictions obtained using tangentially free/slip and tangentially bonded interface assumptions.

Nominal thickness [mm]	ISO f_r measured (mean) [Hz]	FEM free (slip) mean [Hz]	FEM full friction (bonded) mean [Hz]	Error, free (slip)	Error, full friction (bonded)
17	82.2	82.08	85.75	−0.15%	4.32%
22	77.02	77.29	83.16	0.35%	7.98%
27	74.16	72.85	79.78	−1.77%	7.57%
33	70.82	68.55	77.13	−3.20%	8.91%
38	68.52	65.54	76.76	−4.34%	12.03%
43	64.19	62.94	74.13	−1.95%	15.49%
53	58.45	58.55	69.31	0.17%	18.59%
